# The health-promoting experiences of storytellers in group-based digital storytelling workshops: a meta-synthesis of qualitative studies

**DOI:** 10.3389/fdgth.2025.1607897

**Published:** 2025-10-29

**Authors:** Jonathan Dominguez Hernandez, Bernadette Irene Brieskorn, Vanessa Leutenegger, Astrid Krahl, Rachael Eastham, Mark Limmer

**Affiliations:** ^1^ Institute of Midwifery and Reproductive Health, Zurich University of Applied Sciences, Winterthur, Switzerland; ^2^ Department of Health Research, Lancaster University, Lancaster, United Kingdom

**Keywords:** digital storytelling, storytelling, digital narrative, storyteller, digital stories, meta-synthesis, framework analysis, systematic review

## Abstract

**Objective:**

To synthesize qualitative evidence, using the framework analysis method, on how participating in a digital storytelling workshop shapes the storytellers’ health attitudes, values, beliefs, or behaviors.

**Methods:**

We conducted a meta-synthesis using the framework analysis method to generate analytic themes. We searched Medline, CINHAL, SocIndex, Embase, PsycINFO, SciELO, Academic Search Ultimate, Scopus, the Directory of Open Access Journals, and LIVIVO. We used the GRADE-CERQual approach to assess the confidence in the review findings.

**Results:**

We included 25 qualitative studies from six countries representing the experiences of 629 storytellers. Confidence in most review findings was moderate. Storytellers experience digital storytelling workshops as a safe space where they reframe the narratives around their health experiences. This re-storying process extends storytellers’ understanding of their health experiences, affords them a sense of agency and control, and motivates them to use their stories to support others.

**Conclusion:**

We found evidence that digital storytelling enables storytellers to reflect and emotionally engage with the narratives shared in the co-construction of digital stories, resulting in a narrative shift that is likely to be experienced as health-promoting.

**Systematic Review Registration:**

https://www.crd.york.ac.uk/PROSPERO/view/CRD42023478062, PROSPERO CRD42023478062.

## Introduction

A growing body of evidence indicates that digital storytelling as a participatory and community-based approach can provide storytellers with potential health-promoting benefits (1–4). During the digital storytelling process, six to eight individuals who share a common experience work together over three consecutive eight-hour sessions to co-create unique digital stories by sharing and discussing their lived experiences, personal beliefs, and values in response to specific life events (5–7). A trained facilitator guides the storytellers to express and explore their personal experiences in relation to the stories and feedback of fellow participants (5–7). The goal is for storytellers to review their narratives and select and combine music, images, and text with their voiceover to portray compelling stories, engage with the audience, and evoke emotional responses (8). This process results in three to five-minute visual digital narratives eliciting rich, affective, and nuanced insights into the storytellers’ lived experiences (9). The assumption is that as storytellers articulate their lived experiences in a digital story, they gain new insights that could empower them to adopt health-promoting behaviors, values, attitudes, or beliefs (6, 10, 11).

Three phases in the digital storytelling process could explain the mechanisms that drive these potential health-promoting benefits. The story construction phase, which includes writing the script and integrating images, video, and sound, has been found to afford storytellers increased control over their health experiences (12). The collaborative construction approach used during the so-called “story circle” phase, in which the storytellers share their stories with other participants (9), also appears to promote positivity in the group by reducing feelings of social isolation and increasing empathy (2). The final screening phase, where participants screen their stories and discuss their narratives and core elements (9), can also positively affect recipients’ cognition, affect, and health behavior, increase self-efficacy and social support, and positively impact physical and mental health (6, 11, 13). Reports indicate that given these potential benefits, digital storytelling can be used as a learning and capacity-building tool to raise awareness about health issues or to inform and support public health initiatives through advocacy and community empowerment (5, 7, 14). For example, researchers have successfully implemented digital storytelling in youth sexual health promotion (15), to foster dialogue and reduce power imbalances between mental health patients and clinicians (11) or to promote a greater sense of health and well-being in refugee women (16). Digital storytelling has also played a role in youth empowerment in diabetes prevention (17) and has been integrated into participatory efforts for food security and policy development (18). However, it has been argued that any benefits the storytellers may obtain are, at most, anecdotal, coincidental, and a fortunate by-product of the research process because digital storytelling applied in a research setting often has facilitators not trained as therapists, who primarily intend to explore a phenomenon of interest and not how the process influences health attitudes or behaviors (19).

Digital storytelling has previously been subject to review in academic research. However, there is still limited information on the potential health-promoting effects on storytellers because the reviews available did not primarily focus on their experiences (2, 19, 20), narrowed their search to a pediatric (20) or older adult population (21), or did not critically appraise the literature or follow a fully systematic process (3, 22, 23). Therefore, a qualitative evidence synthesis of primary research using an interpretative methodology can add to the current body of knowledge by explaining the potential health-promoting experiences of storytellers. Further, because digital storytelling has been used in diverse contexts, applying a meta-synthesis methodology can enable the integration of contextual nuances that might shape storytellers’ experiences.

## Methods

### Qualitative synthesis methodology

We used the meta-synthesis methodology coined by Stern and Harris (24) and refined by Wals and Downe (22) as an approach rooted in a hermeneutic paradigm that goes beyond the meta-aggregation of the data (23). This approach facilitated an interpretative or meaning-making analysis in generating descriptive themes through an iterative data synthesis process using a framework developed *a priori*. We initially identified each component of this framework and organized and integrated these components into analytic themes, capturing the health-promoting experiences of storytellers (25–27). The ENTREQ checklist items (28) guided our reporting of this meta-synthesis. In addition, some items of the PRISMA statement (29) including the study selection chart or the study characteristics table were used to achieve full transparency in reporting. We registered this meta-synthesis at the inception stage, and there were no deviations from the review protocol (PROSPERO; registration number CRD42023478062).

### Theoretical framework

Salutogenesis was deemed a fitting theoretical framework because it departs from the premise that even in a situation of “illness,” individuals still possess healthy attributes within a continuum between health breakdown and full health (30, 31). We assumed that the digital storytelling process could reveal these healthy attributes and enhance storytellers' sense of coherence (SOC) through the expression and exploration of their personal experiences, fostering health-promoting attitudes, values, beliefs, or behaviors (5–7). Health promotion is this context was defined as the process of empowering people to, individually and collectively, increase control over their health and improve it (32).

### Reflexive note

It was acknowledged from the outset that the reviewers’ experiences, values, and orientations would influence how meaning was derived from the data (33, 34). The principal reviewer had previous experiences participating in digital storytelling workshops and continuously reflected, critiqued, and evaluated his prior beliefs when synthesizing and interpreting the textual data. We consciously and deliberately looked for evidence that challenged these beliefs to avoid overemphasizing data aligned with pre-existing viewpoints. In addition, discussions with the other reviewers questioned how much the principal reviewer could have imposed meaning on the data items. None of the other reviewers had previously participated in a digital storytelling workshop, and as midwives and academics, this was their first contact with digital storytelling. We aimed to minimize the over-influence of the principal reviewer through this member reflection process and ensure the credibility of the findings (35).

### Inclusion criteria

We included studies exploring the health-promoting experiences of storytellers participating in a digital storytelling workshop. We defined digital storytelling as a collaborative, participative, and group-based approach to co-create three- to five-minute digital stories portraying participants’ experiences of health. We considered studies for inclusion if they employed qualitative methods and included the qualitative components of mixed-methods studies. The inclusion criteria and their rationale are detailed in Table 1.

**Table 1:** Inclusion criteria.

SPIDER	Inclusion criteria	Exclusion criteria	Rationale
Sample	All populations, including pediatric populations	N/A^a^	Digital storytelling is accessible to all populations with the ability to write, read, and use a computer. This can include the majority of the pediatric population. Digital storytelling is a facilitated process that can be adapted to all populations. In addition, digital storytelling experiences are unique for the storytellers. Including a wider population can provide a more general understanding of various health-promoting experiences.
Phenomenon of interest	The health-promoting potential of digital storytelling on the storyteller in a health context	Digital storytelling outside the health context	Digital storytelling has been widely used as a pedagogical approach in education and as an instrument for persuasion in marketing. It was essential to differentiate these studies from those addressing health issues.
Design	Interviews, focus groups, observations, case studies or action research	Surveys, online surveys	Measuring lived experience with an instrument remains a challenge. Researchers who have employed standard instruments to measure experiences of digital storytelling participation have not found evidence of an effect (54).
Evaluation	Health attitudes, values, beliefs, behaviors	N/A	Research evidence indicates that digital storytelling can lead to storytellers replacing their health narratives with health-promoting attitudes, values, beliefs or behaviors (82).
Research type	Qualitative or the qualitative components of mixed-method studies	Observational (Cross-sectional, etc.) experimental and quasi-experimental studies	To ensure a comprehensive representation of the available qualitative evidence. In addition, it provides richer and more in-depth data on the storytellers’ experiences.
Language	The search strategy was developed in English, German, and Spanish, but no language restrictions were applied during the search or screening process.	N/A	Based on the principal reviewer's language competence. The titles and abstracts of articles in other languages were translated using artificial intelligence through DeepL (translator function).
Type of publication	Primary research, including published peer-reviewed and grey literature (such as dissertations or other type of reports of primary qualitative research)	Non-primary research such as editorials, letters to the editor, reviews, case reports, and case series	Primary qualitative research offers rich and in-depth data, and grey literature (such as dissertations and reports) can contribute to more diverse viewpoints and nuances.

The health-promoting experiences of storytellers participating in group-based digital storytelling workshops (Meta-synthesis, Switzerland, 2024).

^a^ Not applicable.

### Data sources

We searched Medline (Ovid), CINHAL (Ebsco), SocIndex (Ebsco), Embase (Ovid), and PsycINFO (Ebsco) in November 2023. Additionally, a search in the SciELO, Academic Search Ultimate (Ebsco), Scopus, the Directory of Open Access Journals (DOAJ), and LIVIVO databases expanded the search for potential articles, particularly in Spanish, German, and the global south. The global reach of digital storytelling demanded the inclusion of articles in other languages. We also reviewed the lists of references from the included studies and retrieved grey literature using the Bielefeld Academic Search Engine (BASE).

### Search strategy

The search strategy included Boolean and proximity operators, subject headings (MeSH), word searching (free text), and the use of spelling (truncation), syntax, line numbers, and publication-type filters to retrieve qualitative and mixed methods articles. We applied free-text terms to cover unavailable subject headings (36). We initially developed the search strategy in Medline (via Ovid) using keywords such as “digital” and “stories,” “storytelling,” “narratives,” “interviews,” or “qualitative” and adapted the search to fit other databases (see Supplementary Appendix A1). Search terms in English were then translated into German and Spanish, although the German terms proved unhelpful in retrieving articles (37).

### Study screening

Two reviewers screened the retrieved articles independently. A third independent reviewer resolved disagreements arising during the stages of the screening process (38), including an initial title and abstract review guided by a screening tool, illustrating the inclusion criteria and assisting the reviewers with inclusion decisions. An initial piloting phase of this tool with twenty-five articles assisted reviewers in ascertaining its applicability and ability to maximize inter-rater reliability (39). All retrieved studies were exported after deduplication from Endnote to Covidence (40). We used the same screening process to screen the full-text articles of included studies.

### Quality of included studies

We used the critical appraisal skills program (CASP) tool to evaluate the methodological rigor of the included studies (41). The principal reviewer independently assessed the quality of all included studies, and three reviewers performed the second independent assessment, each evaluating one-third of the included studies. Conflicts were resolved through consensus (42). We did not exclude any articles based on quality rating, but as a form of sensitivity analysis, we explored how removing the findings from weaker studies would influence the analytic themes (43).

### Data extraction

We extracted data items such as the philosophical position, theoretical assumptions, research methods, contextual nuances, and findings. Using an adapted version of the Joanna Briggs Institute's (JBI) instrument (44), two reviewers piloted the tool with a purposive sample of two articles (45). The goal was to limit interpretation and selection errors (46). The principal reviewer independently extracted all data items from all included studies, and three reviewers independently extracted the data from one-third of the included studies.

### Data synthesis

A framework analysis approach was used to synthesize the data. Two index papers of the best quality were selected to develop the initial framework. Two reviewers independently open-coded the findings using NVivo and created a matrix containing the initial codes and descriptive themes. We discussed each coded section to determine its significance and how it could answer the research question. Conflicts were resolved by returning to the index studies to agree on the most suitable theme. We entered the final themes into a spreadsheet to organize and map the data against this framework (see Supplementary Appendix A2). The initial framework was refined iteratively as new themes emerged from other studies. We created or allocated new themes to existing categories using the constant comparative approach. If a new theme emerged, we assigned a name (a metaphor) and compared it with those included in the initial framework (47). The quality of the included studies assisted reviewers in determining the emphasis of new emerging themes in contrast with those within the framework and their closeness to the theoretical framework. For instance, new themes from high-quality studies replaced or modified existing ones (see Supplementary Appendix A2).

After coding and mapping the data, we began interpretation, ensuring the original themes’ meaning was preserved (22). We used the framework to cluster metaphors and reach a consensus on the descriptive themes. Participant quotations for each review finding illustrate the final themes. We assessed the level of confidence in the review findings using the GRADE-CERQual approach, considering methodological limitations, coherence, that is, how well the data supported the review findings, their adequacy or the degree of richness and quantity of the data, and the relevance of the data concerning the specified context of the meta-synthesis (48). The assessments of these four components collectively contributed to a level of confidence ranging from high to very low (49) (see Supplementary Appendix A3). We defined a review finding as an analytical result that describes a phenomenon or an aspect of a phenomenon based on data from primary studies (50).

## Results

**Figure 1:**
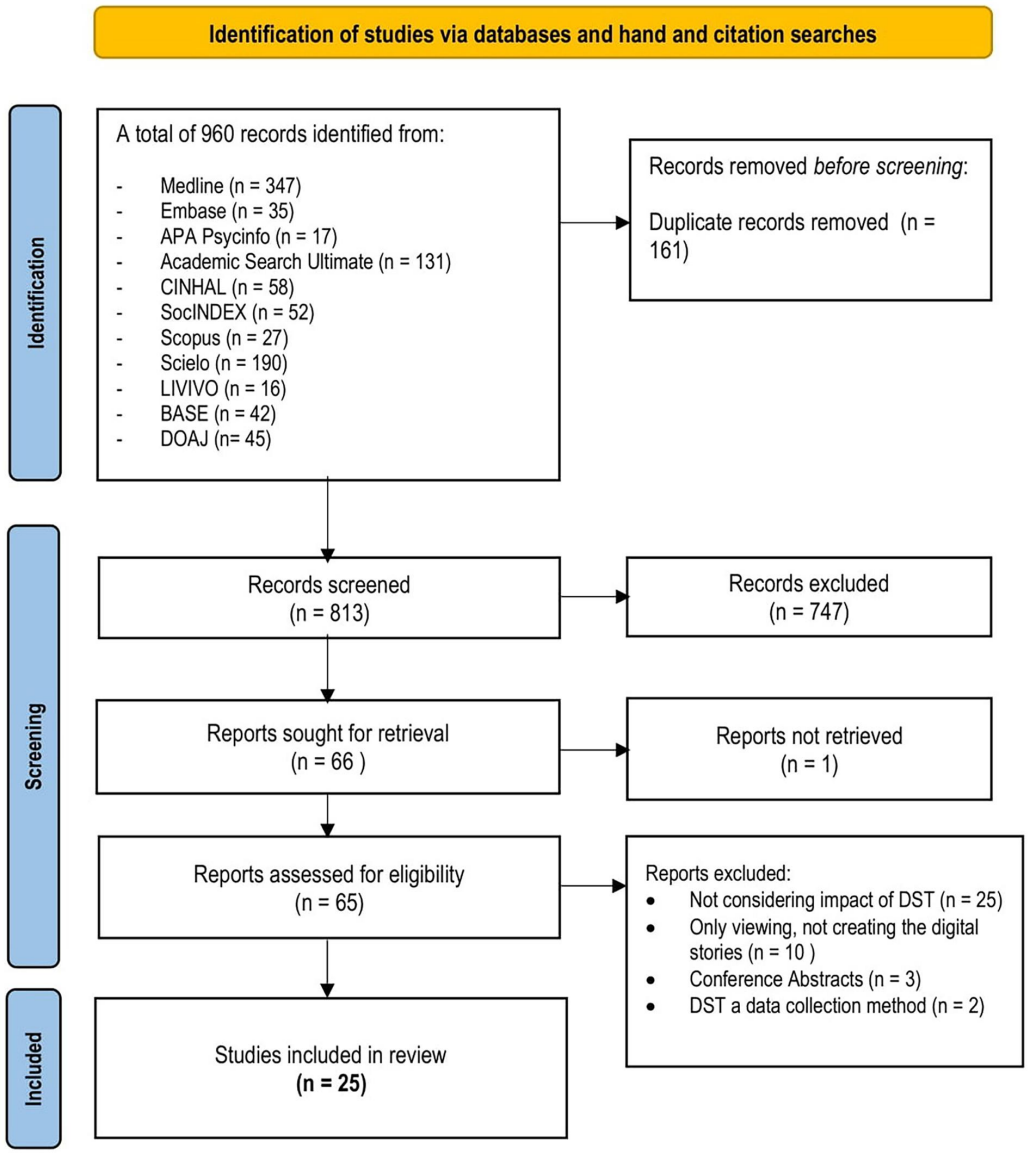
PRISMA flow diagram of the study selection process (Switzerland, 2024).

The review identified 974 records from electronic databases and hand and citation searching. After removing 161 duplicates, the reviewers screened the titles and abstracts of 813 articles. This led to the final inclusion of 25 peer-reviewed qualitative and mixed-methods articles exploring storytellers’ experiences (Figure 1).

### Studies characteristics

The included studies were conducted in the United States, Canada, Australia, New Zealand, Malawi and Zimbabwe. The sample sizes ranged from two participants to a sample of 342, making a total of 629 participants. Most studies used StoryCenter's (51) approach to digital storytelling or the approach of the Creative Narrations organization (52). A detailed description of the characteristics of the included studies can be found in Table 2.

**Table 2:** Characteristics of the included studies.

Author(s), Year	Country	Population	Sample size	Context/Setting	Aim(s) or research question(s)	Philosophical/Theoretical underpinnings	Study design	Digital storytelling approach	Data collection	Analytic process	Reflexivity
Beltrán and Begun 2014 (5)	New Zealand	Māori community (20–45 years)	*n* = 6	University Campus	To facilitate dialogue and use digital storytelling to interrupt contemporary challenges often faced by Indigenous communities	Historical trauma theory	Community-based participatory inquiry	StoryCenter	In-depth qualitative interviews	Thematic analysis	Yes
Boydell et al. 2018 (83)	Canada	Young people (16–23 years) with the first episode of psychosis	*n* = 9	Early intervention clinic rural community	To produce individual digital stories describing how to manage psychosis in everyday life	The social world “does not consist of separate things. but of relationships.”	Community-based participatory inquiry	StoryCenter	Participant observation informal interviewing and reflexive field notes	Not described	No
Briant et al. 2016 (10)	USA	Working-class Hispanics of Mexican origin	*n* = 11	Cancer Research Center	To explore if digital storytelling could be a culturally relevant tool for Hispanics/ Latinos of Mexican origin to share their experiences with cancer or other diseases.	Transportation theory	Community-based participatory action research and a narrative framework	Creative Narrations digital storytelling	Semi-structured face-to-face interviews	Thematic analysis	No
De Vecchi et al. 2017 (11)	Australia	Mental health consumers and clinicians	*n* = 11	Rural psychiatric service	To explore participation in digital storytelling and investigate the potential of digital storytelling as a research method.	Interpretative paradigm	Instrumental Case study and process evaluation	StoryCenter	Semi-structured Interviews	Not described	No
DiFulvio et al. 2016 (84)	USA	Puerto Rican Latinas (15–21 years)	*n* = 30	New England City	To assess the effectiveness of the digital storytelling process in creating positive changes in participants’ sexual attitudes or values, self-esteem, social support, and empowerment (including self-efficacy, sense of hope for the future, and community activism).	Not reported	Mixed-methods	StoryCenter	Qualitative part: Open Ended questions	Not reported	No
Dixon and Isaac 2023 (85)	USA	Black African-American women, cisgender, queer, and middle-class, public health doctoral students	*n* = 2	Weekly Zoom meetings	To explore how the intersection of racism, sexism, and socio-cultural expectations shape BFRB experiences.	Critical Narrative Archaeology of Self (AOS)	Autoethnography	StoryCenter	Digital storytelling process and online discussions	Content analysis	Yes
Ferrari et al. 2015 (7)	Canada	Persons of African, Caribbean, and European descent	*n* = 15	Toronto, Ontario,	To examine how the process of making their digital stories can be “therapeutic” or “cathartic.	Not reported	Not reported	StoryCenter	workshop evaluation and 11 of them a survey	Thematic analysis	No
Fiddian-Green et al. 2017 (86)	USA	Puerto Rican Latinas (15–21 years)	*n* = 10	New England City	To examine and delineate salient cultural paradigms of sexuality.	Constructivist grounded theory	Case-Study	StoryCenter	Semi structured interviews	Content, discourse analysis	No
Goodman 2019 (87)	Canada	Drug users (25–45 years)	*n* = 30	Drug recovery clinic	To help transform widespread assumptions and prejudices about long-term heroin users	Not reported	Not described.	StoryCenter	Interviews	Descriptive, digital stories	Yes
Gubrium et al. 2016 (54)	USA	Puerto Rican Latinas (15–21 years)	*n* = 6	New England City	To know if the digital storytelling process affected a participant's self-esteem, sense of empowerment, social support, or sexual attitudes or behaviors.	Constructivist grounded theory	Mixed-methods	StoryCenter	Story transcripts, Field notes, observation, follow-up semi-structured interviews	Using code matrix and three node structure	Yes
Gubrium et al. 2019 (53)	USA	Puerto Rican Latinas (15–21 years)	*n* = 30	Offices of a community-based organization	To collaboratively interrogate and address experiences of interpersonal and structural violence and embodied trauma.	Critical Narrative	Digital storytelling as a research method	StoryCenter	Digital storytelling, field notes and follow-up interviews	Intertextual transcription method, content analysis, contextual analysis	Yes
Howard et al. 2023 (55)	Canada	People with endometriosis	*n* = 6	Online workshops via Zoom.	To assess the feasibility of story co-creation and sharing.	Not reported.	Qualitative methods	StoryCenter	Reflective journals	Qualitative interpretive description	Yes
Jun et al. 2022 (88)	USA	Nurses from a not-for-profit public-health organization	*n* = 13	Workshops offered within the nursing organization	To evaluate the first-person experiences of attending in-person digital storytelling workshops.	Not reported	Descriptive qualitative method	StoryCenter	Semi-structured phone interviews	Thematic analysis	Yes
Kim et al. 2021 (89)	USA	Adults (18 or older) who received a hematopoietic cell transplant	*n* = 4	Not known	To explore experiences of participating in a 3-day digital storytelling workshop	Narrative theoretical model/social cognitive theory/constructivist grounded theory	Descriptive qualitative method	StoryCenter	Workshop evaluation questionnaire	Content analysis	No
Kim et al. 2023 (90)	USA	Vietnamese or Korean American women (18 or older)	*n* = 8	Virtual digital storytelling via Zoom	To assess feasibility, acceptability and in-depth analysis of the mother's cultural experience of HPV	Constructivist grounded theory	Mixed methods	StoryCenter	Questionnaire, field notes, web-bsed workshop activtivities	Thematic analysis	No
Laing et al. 2017 (91)	Canada	Children and young adults cancer survivors (5–19 years)	*n* = 16	Childreńs hospital	To determine how digital stories might be effective therapeutic tools	Hermeneutics	Not reported	StoryCenter	Semi-structured interviews	Hermeneutic	No
Laing et al. 2019 (92)	Canada	Adult (18 or older) patients with cancer	*n* = 10	Outpatient cancer treatment center	To discover how the creation of a digital story affects adult patients with cancer	Hermeneutics	Qualitative, interpretive methodology	Two-hour sessions with assistant trained in digital storytelling	Semi structured interviews face to face and telephone	Hermeneutics	No
Lamarre & Rice 2016 (93)	Canada	Young Women with Eating Disorder Recovery	*n* = 10	Not described	To illustrate the possibilities of a guided practice of digital storytelling for critical arts-informed research processes	Critical feminism/theoretical frame (post-structuralist)	Narrative	StoryCenter	Digital stories	Visual analysis	No
Lenette & Boddy 2013 (16)	Australia	Women with refugee backgrounds	*n* = 3	Not reported	To explore women's experiences of being a refugee	Feminism	Visual ethnography	Not described.	Photovoice, Photo-eliciting and digital storytelling, in-depth interviews	Visual ethnography	No
Martin et al. 2019 (94)	Canada	Young women exposed to dating violence	*n* = 6	University campus	To explore the impact of learning digital storytelling skills to promote change and interrupt the cycle of abuse.	Feminist, qualitative, and arts-based research	Digital storytelling as a research method	StoryCenter	Focus group	Not described	No
Njeru et al. 2015 (95)	USA	Somali and Spanish-speaking patients with type II diabetes	*n* = 8	Healthy Community Partnership centre	To develop a diabetes digital storytelling intervention with and for immigrant and refugee populations.	Social Cognitive Theory, Narrative Theory, and social construction theory	Digital storytelling as a research method	StoryCenter	Digital Stories	Not described	No
Nyirenda et al. 2022 (96)	Malawi	Residents from Bangwe and Ndirande	*n* = 26	Bangwe and Ndirande, Malawi	To assess if digital storytelling could be used to explore community perspectives on health concerns.	Gaventa's theories of power and participation	Digital storytelling as a research method	Similar to the StoryCenter approach	Digital Stories	Not described	No
Paterno et al. 2018 (97)	USA	Peer mentors in recovery from SUD	*n* = 5	Community site in a rural county in New England	To assess the feasibility of using digital storytelling with peer mentors.	Not described	Digital storytelling as a research method	StoryCenter	In-depth, semi structured follow-up interviews	Thematic analysis	No
Wexler et al. 2013 (98)	USA	Young people from 12 rural villages in Northwest Alaska	*n* = 342	Rural sparse populated areas of Alaska	To use digital storytelling as a health promotion strategy within a PYD approach.	Freirian Model	Mixed-methods	StoryCenter	Interviews after workshop	Coding but not further description	No
Willis et al. 2014 (99)	Zimbabwe	Young people with HIV (8-22 years)	*n* = 12	Africaid's Zvandiri program	To evaluate the digital storytelling process as a therapeutic approach	Social constructionism	Qualitative methods	StoryCenter	Focus groups digital stories, field notes,	Thematic analysis	No

The health-promoting experiences of storytellers participating in group-based digital storytelling workshops (Meta-synthesis, Switzerland, 2024).

### Methodological rigor

There were some concerns with the overall methodological rigor of the included studies, with only three studies being rated as having no methodological weaknesses that would impact the transferability of the results (53–55). In this meta-synthesis, transferability refers to the extent to which study findings can be subjected to de-contextualization and abstraction to generalize the emerging themes to other situations (56). The biggest issue of concern was the lack of reflexivity, with 14 studies either poorly addressing or not reporting any aspects of personal, interpersonal, methodological, or contextual reflexivity. A detailed description of our assessments is presented in Table 3.

**Table 3:** Methodological rigor of the included studies.

Author(s), Year	CASP tool questions								
	Q1	Q2	Q3	Q4	Q5	Q6	Q7	Q8	Q9
Beltrán and Begun 2014 (5)	Yes	Yes	Yes	No	Yes	Yes	Can't tell	Can't tell	Yes
Boydell et al. 2018 (86)	No	Yes	Yes	Can't tell	Yes	No	No	Can't tell	No
Briant et al. 2016 (10)	Yes	Yes	Yes	Yes	Yes	No	No	Yes	Yes
De Vecchi et al. 2017 (11)	Yes	Yes	Yes	No	Yes	Can't tell	Yes	Can't tell	Yes
DiFulvio et al. 2016 (88)	Yes	Yes	Yes	Yes	Yes	No	Yes	No	Yes
Dixon and Isaac 2023 (85)	Yes	Yes	Yes	Can't tell	Can't tell	Yes	Can't tell	Yes	Yes
Ferrari et al. 2015 (7)	No	Can't tell	Yes	Can't tell	Yes	No	No	Cańt tell	Yes
Fiddian-Green et al. 2017 (86)	Yes	Yes	Yes	Yes	Yes	Can't tell	Yes	Yes	Yes
Goodman 2019 (87)	No	Yes	Can't tell	Yes	Yes	Yes	Yes	No	Yes
Gubrium et al. 2016 (54)	Yes	Yes	Yes	Yes	Yes	Yes	Yes	Yes	Yes
Gubrium et al. 2019 (53)	Yes	Yes	Yes	Yes	Yes	Yes	Yes	Yes	Yes
Howard et al. 2023 (55)	Yes	Yes	Yes	Yes	Yes	Yes	Yes	Yes	Yes
Jun et al. 2022 (88)	Yes	Yes	Yes	No	Yes	Yes	Yes	Yes	Yes
Kim et al. 2021 (89)	Yes	Yes	Yes	No	No	Can't tell	Yes	Yes	Yes
Kim et al. 2023 (90)	Yes	Yes	Yes	Yes	Yes	Can't tell	Yes	Yes	Yes
Laing et al. 2017 (91)	Yes	Yes	Yes	Yes	Yes	Can't tell	Yes	No	Yes
Laing et al. 2019 (92)	Yes	Yes	Yes	Yes	Yes	Can't tell	Yes	Can't tell	Yes
Lamarre & Rice 2016 (93)	Yes	Yes	Can't tell	Can't tell	Yes	Yes	No	Can't tell	Yes
Lenette & Boddy 2013 (16)	No	Yes	Yes	Yes	Yes	Can't tell	Yes	Can't tell	Yes
Martin et al. 2019 (94)	Can't tell	Yes	Yes	Can't tell	Yes	Can't tell	Yes	Yes	No
Njeru et al. 2015 (95)	Yes	Yes	Yes	No	Yes	Can't tell	No	Can't tell	Yes
Nyirenda et al. 2022 (96)	Yes	Yes	Yes	Yes	Can't tell	Can't tell	Yes	Can't tell	No
Paterno et al. 2018 (97)	Yes	Yes	Yes	No	Yes	Can't tell	Yes	Can't tell	Yes
Wexler et al. 2013 (98)	Yes	No	Yes	Yes	Yes	Can't tell	Yes	No	No
Willis et al. 2014 (99)	Yes	Yes	Yes	No	Yes	Can't tell	Yes	Yes	No

The health-promoting experiences of storytellers participating in group-based digital storytelling workshops (Meta-synthesis, Switzerland, 2024).

### Synthesis output

This meta-synthesis generated 12 descriptive themes (review findings), of which nine were graded with moderate confidence, indicating that these review findings are likely a reasonable representation of the storytellers’ health-promoting experiences. Two themes were graded with high confidence and a single theme with low confidence, making them highly likely or possibly a reasonable representation of the storytellers’ experiences, respectively. From these descriptive themes, we derived four analytical themes:—“Re-Storying Lived Experience,” “The Ripple Effect Of Digital Storytelling,” “Investing And Processing Emotions,” And “Owning The Script.” The final framework and its associated CERQual gradings are presented in Table 4.

**Table 4:** Framework and associated CERQual gradings.

Descriptive themes (Review Findings)	Studies contributing to the review finding	GRADE-CERQual Assessment of confidence	Example of Supporting data	Analytical theme
**Overcoming vulnerability**—Entering the digital storytelling space often generates initial fears and apprehensions triggered by the prospect of sharing sensitive and intimate stories about health issues with strangers. Storytellers fear being judged or misunderstood and grapple with the uncertainty of how their stories unfold. Storytellers cautiously navigate the digital storytelling process, but this sense of vulnerability fades as they share their stories. Storytellers recognize their peerś bravery, inspiring them to share their truths and reinforcing the belief that their stories must be told.	11 Studies (11, 53–55, 83, 84, 86, 88, 90, 91, 96)	Moderate confidence	“In the beginning, I was really, really anxious about it, but even halfway through the first session, that anxiety definitely decreased just at how welcoming and accepting and just how common it felt our experience was. We had this like connection that was beyond just Endo, having Endo. It was really great” (55). “I think there might have been one person that wasn't quite ready, maybe being vulnerable. But it might be a little bit easier if they know that other people on their team are participating. Being around complete strangers for three days is kind of terrifying” (88).	Re-storying lived experience
**Deliberate sense-making**—Storytellers make sense of their lived experiences of health, illness and trauma as they write their scripts, collect visual and auditory material to illustrate their stories and share them with fellow storytellers. This process provides a space for deliberate reflection, finding meaning from their experiences, and capturing their emotional state. Some storytellers find new meaning in their experiences and value the opportunity to learn more about themselves as individuals and community members through this deliberate process.	14 Studies (5, 7, 10, 11, 54, 55, 83, 87, 88, 91, 92, 95, 97, 98)	Moderate confidence	“It sort of forces you to reﬂect on things that maybe you don't sit down and do every day, right? It's not like you go, “Today I'm going to think about this.” This is kind of an opportunity that forces you into something like that. It made me think about things that maybe I didn't want to, or hadn't very much, you know?” (97). So it wasn't easy, to share my story, but I guess it's being able to step back from those immediate moments, and look at them a little more objectively. It really helps to make sense of things, make meaning, and see how your story is connected to other pieces of your life. we're looking for meaning, and I just wanted to find some meaning in what I'd been going through” (87).	
**Shaping or replacing existing narratives**— Storytellers undergo a profound shift in perspective as they construct their stories, resulting in the altering, reshaping, or even replacing the narratives they initially brought into the digital storytelling process. Storytellers uncover previously unknown connections to other aspects of their lives by re-examining their own beliefs, perspectives, and lived experiences as they listen to the stories of others. Some storytellers view it as an opportunity to challenge and correct existing, socially accepted narratives and discourses and as an accomplishment.	16 Studies (5, 7, 10, 11, 54, 55, 83–85, 87, 91–93, 96, 98, 99)	Low confidence	“Life-changing stuff. You can see it. You can hear it in people's narratives. It's about talking through their story. You can hear the growth and the de-cluttering. Yeah, it's almost like watching a flower, like a peony rose… beautiful flowers…and it's just like watching them burst into life” (5). "I no longer want to put pressure on myself to live up to the definitions of those concepts [of Anorexia], whether externally or internally imposed. That's kind of the point” (93).	
**Sharing and connecting in a safe space**—The digital storytelling process creates a safe and supportive space, an environment of mutual respect and understanding, where storytellers are empowered to speak freely about their experiences of health, even about their most guarded secrets, as they develop a sense of connection with other storytellers.	7 Studies (5, 10, 11, 54, 55, 89, 97)	Moderate confidence	“It was also nice to get so much support, because I don't know if I would've shared that story had I not had the environment being the way it was, because it was so safe, …it was kind of like a powwow, where we all came together and really understood where each other was coming from” (5). “I think it can allow someone to enter that space with you and someone might not have that lived experience, but that story you're telling might map onto some other experiences they've had..you can create a bit of shared space and shared understanding” (11).	The ripple effect of digital storytelling
**From empathy to compassion**: Listening to other storytellers' stories generates an empathetic response driven by learning about their shared experiences, motivating storytellers to act and mutually support each other. Some storytellers gain an awareness that they are part of something bigger and feel that their stories can help both fellow storytellers and those beyond the digital storytelling workshop. They feel compelled to tell their stories to help others.	14 Studies (5, 10, 11, 54, 55, 83, 84, 87, 89–91, 97–99)	Moderate confidence	“I think it was great when everyone talked and supported each other. Number one, if you heard their story, you understood where they were coming from because you can actually feel it and connect to those who experienced the same thing” (89). “A lot of people down here, they get in this rut, you know, and it just seems like there's no way out. I want to say that there is a way out and it can be very rewarding” (87).	
**Sharing stories brings comfort**—During digital storytelling, storytellers learn that they are not alone, which brings them comfort. Knowing that others have similar experiences validates the storytellers' feelings and emotions, and storytellers find common ground in their experiences, generating a sense of group hope and togetherness.	8 Studies (5, 7, 10, 53–55, 83, 88)	Moderate confidence	“It reminded me that I wasn't alone. Not only are there others who share my experiences, but [they] share my view of the experiences.” (7). “I find myself relating to every single story. I have a story within that story … and then I think maybe I need to get to know someone else.” (91). “I find myself relating to every single story. I have a story within that story … and then I think maybe I need to get to know someone else” (91).	
**Increased Sense of Community Belonging**—Learning that others go through similar situations generates a strong bond between the storytellers, inspiring a commitment beyond the workshops. Storytellers demonstrate a commitment to sustaining and growing their newly gained community.	10 Studies (5, 10, 11, 54, 55, 88, 89, 94, 97, 98)	High confidence	“I was very moved by [name] story as so much of my own is captured in her words. Again, I felt a sense of connection and of a deep understanding from my fellow participants, which was really beautiful and made me want to think about how to connect further with these women or others who have Endo” (55). “[The DST] allowed me to get to know my peers on a more intimate level. Even though we worked together, there was so much we didn't know about each other and, I mean, their videos brought me to tears, and I felt like I really got to know them” (97).	
**Experiencing emotional resonance**—The digital stories portray emotions and feelings that resonate with the storytellers. As storytellers engage with the stories, they recognize and connect with these emotions as they realize they all share similar emotional experiences.	8 Studies (5, 11, 54, 55, 88, 91, 92, 94)	Moderate confidence	“…I found it challenging because some of the stories were.. emotionally resonating..you felt for what people go through, what's behind that outer shell that you show to the world” (11). “Maybe it's the shared emotions like sometimes the release of the emotions …. Being heard and being seen as a human, not just as a nurse … Our whole job is all about secondary trauma; hearing stories that are so challenging that you have to be able to put that somewhere ….” (88).	Investing and processing emotions
**Harnessing the amplified emotions**—Triggered by the evoked emotional resonance, storytellers experience a heightened emotional awareness as they write and produce their digital stories and listen to the stories of others. This is experienced as an opportunity to work and process these emotional burdens and open up to sharing things that otherwise, in other contexts, they would not share. The emotions drive storytellers to construct stories that portray “the rawness of their experiences”.	8 Studies (5, 55, 84, 87, 89, 91, 92, 94, 97)	Moderate confidence	“I was surprised at how much emotional I was when I went over my real story. I never thought of once any of the feelings that I was having. This workshop opens me up to realize the value of talking and sharing emotions. Now I am sharing my emotions with my family” (89). “The making of the video was out of anger, like you know, I just wanted to get it out so bad. And yeah, it's a little hard to explain.…Like I just let it all spill out on the video, kind of thing…I was really angry when I was looking at those pictures” (89). “Like, your choice really is to let [the emotions] crush you or not. And when your choice is to not, then you have no choice but to stand up and make sure you are in the best frame of mind, which means you have to do something with those emotions” (92).	
**Coming to terms with experiences of health**— Storytellers experience a sense of relief from their emotional burdens, feel liberated from memories of adverse experiences, and feel at peace, having shared their stories with fellow storytellers who understand their pain and suffering. Some storytellers refer to the process as therapeutic and cathartic because it helps them confront and process their emotions and feel healed, having achieved some form of personal growth.	11 Studies (5, 7, 10, 54, 55, 83, 84, 87, 88, 91, 93)	Moderate confidence	“I grew. I was liberated and at peace. I'm at peace with myself in a way that I've not been for…I don't know…probably forever…by getting [the story] out, because I harboured it for so long. That's not a healthy way to be. It really makes…well, talking about health and trauma… it is medicine. That's the best way I can say it” (5). “[I] feel no more guilt now that my story is right,” and others who spoke of the value of “open[ing] up” and “let[ting] go” of “things we've kept inside” (84).	
**Increased sense of control over their experiences**— As storytellers construct their stories, they go through a process of self-discovery that appears to build their confidence. They feel they have some influence or power over their circumstances. This is amplified by the collaborative approach to story development, which moves them to co-create stories that either challenge existing narratives and discourses about their lived experiences or send a message to help others.	13 Studies (5, 7, 10, 53–55, 83, 84, 87, 89, 91, 97, 99)	High confidence	“…I just think that when you look at someone who's like you—looks like you—and they tell a story that is similar to yours, and they've come through it and they are now putting voice to it, and now they're not hurting, it gives you hope. It gives you hope that, ‘Well, what if I put a voice to my story?” (5). “…it is ‘brave of us to share our stories—it teaches us as mothers, parents—that these are the things we can teach our children—about domestic violence, birth control. They don't have to go through the same thing. This brought us together as mothers and people” (53). “I can now take control of my life, I have kissed away the fear and frustration” (99). “Through support, I feel conﬁdent and able to make informed choices. I am now conﬁdent, independent and most of all ever smiling” (99).	Owning the Script
**Gaining Agency**—After completing the digital story, storytellers recognize that the stories can potentially resonate with others. They acquire a sense of purpose and express their desire to use their stories to improve the lives of those with similar experiences.	8 Studies (5, 10, 54, 55, 89, 92, 97, 99)	Moderate confidence	“I definitely want to take my story and show it to my iwi, my family, and I want to see if they would be interested in doing some work of this nature. I feel like I've been given something that will help me help someone in a very simple way…” (5). “…this digital recording it's very important because we're making it known…we are letting our community know that many things can be prevented.” (10). I was feeling so lost…and then now I just kind of feel filled with so much purpose. Just being able to share your story with someone who wants to listen is…well…I, I think we often don't realize that something simple and little like that can be so helpful” (89).	

The health-promoting experiences of storytellers participating in group-based digital storytelling workshops (Meta-synthesis, Switzerland, 2024).

#### Re-storying lived experience

Participation in a digital storytelling workshop prompts storytellers to alter, reshape, or even replace their narratives concerning their lived experiences of health with new or more positive ones. We assumed the distinction between stories, the specific tales shared about their experiences, and narratives, the broader contextual or social resources storytellers draw upon to construct their stories (57). Storytellers reconsider their experiences as they listen to and share stories with fellow participants, triggering a deliberate sense-making process that helps storytellers make new connections to other aspects of their lives and gain new narratives. They use these new narratives to create their digital stories, often leading to telling a different story than initially intended. This re-storying becomes an accomplishment and reshapes their understanding of their experiences and broader discourses around their health. Initially, storytellers feel vulnerable sharing sensitive and intimate stories about their health with strangers, worrying about being judged or misunderstood and about how their stories will unfold. However, this apprehension fades as they listen to the stories of others, finding inspiration and reinforcing the belief that their stories must be told.

#### The ripple effect of digital storytelling

Despite their initial apprehension, storytellers experience digital storytelling as a safe, supportive, health-promoting space. As they listen to the stories, they learn that all share similar experiences, creating an environment of understanding where they feel empowered to share their health experiences openly. The stories resonate with the storytellers and make them feel connected, validated, and comforted by learning that they are not alone. It is a ripple effect, which begins with the storytellers becoming story recipients. Engaging with the stories provokes an empathic response from fellow storytellers, triggering emotional and physical responses such as expressing admiration, feeling inspired or moved, or even embracing the narrator to comfort them. These responses, in turn, foster a sense of community belonging, make participants feel hopeful, and inspire a commitment beyond the workshop. Storytellers realize they are part of something bigger and feel compelled to tell their stories to help others. This shift from empathy to compassion involves a deep connection with the storytellers’ experiences of health, which requires a personal knowing of those experiences, evoking caring actions that bring comfort (58). Compassion here is a deliberate process in which storytellers treat others as they wish to be treated themselves.

#### Investing and processing emotions

The storytellers share more than similar stories and experiences. They vividly recognize the emotions described and portrayed in the stories shared as some of their own. Learning about and experiencing the emotions of others heightens the storytellers’ awareness of their own emotional state. Storytellers harness this intensified emotional awareness and use it to work and process their emotional burdens into their digital stories. Their emotional responses drive storytellers to open up to sharing things they would not express elsewhere and co-construct compelling stories that portray the authenticity and rawness of their experiences. Some storytellers refer to this process as therapeutic and cathartic because they experience some relief from their emotional burdens by sharing their stories with empathetic listeners who understand what they are going through.

#### Owning the script

The digital storytelling process affords storytellers the confidence to steer the narratives around their experiences. They acquire a sense of control over their personal narratives by articulating them into a digital story. Some storytellers view the process as an opportunity to challenge existing narratives and discourses about living with a particular condition or trauma. They deliberately seek to convey a message, a counternarrative, a first-hand and accurate account of their experiences. They aim to share their truths with others, even if it means evoking uncomfortable responses from recipients. Upon completing their digital stories, storytellers often recognize the potential impact of their narratives on others. Through this process, storytellers gain agency and are motivated to transform their digital stories into powerful tools to support others.

## Discussion

This meta-synthesis used the framework analysis method to synthesize the health-promoting experiences of storytellers participating in a group-based digital storytelling workshop. Our GRADE-CERQual assessment of the findings provided evidence of moderate confidence that co-constructing digital stories about health experiences can shape the storytellers’ attitudes, values, beliefs, or behaviors. This is evidenced by four analytic themes containing health-promoting attributes likely to reasonably represent the storytellers’ experiences.

To gain an insight into how these four analytic themes contain health-promoting attributes, we employed the salutogenic lens underpinning this meta-synthesis. The first analytic theme — Re-storying Lived Experience — illustrates a narrative shift that can strengthen the storytellers’ sense of coherence (SOC). The SOC expresses an individual’s ability to successfully respond to a stressful situation when it is deemed worth dealing with. It is essential for developing and maintaining health, as a stronger SOC generally correlates with better-perceived health (59–61). The SOC can be strengthened by reflecting on stressful situations and identifying appropriate resources (62). We explained that the narrative shift results from a reflection process triggered by the storytellers’ deep engagement with the shared narratives. Although the included studies do not always detail the narratives` characteristics shared between the storytellers, it is plausible that storytellers are exposed to various narrative structures and contents that can have health-promoting attributes during the storytelling process. For instance, research evidence has identified key often shared mental health recovery narrative forms, including narratives of escape, such as resisting stigma, or narratives of enlightenment, where the storyteller views the illness or trauma as ultimately positive (63). There is strong evidence suggesting that narratives of this kind can be health-promoting by reducing stigma, helping individuals better understand mental illness and how to recover, or validating their experiences (13). Exposure to the narratives of individuals who have faced or overcome real-life health challenges has also been found to help storytellers (64). Thus, the various narratives forming the basis of the shared digital stories can be considered resources storytellers draw upon to re-examine or revise their own narratives, steering them towards health-promoting forms, content, or structures. From a salutogenic perspective, the more resources (generalized or specific) storytellers possess to appraise or reflect on their experiences, the more likely they are to shift their narratives and perceive them as more manageable and meaningful, strengthening their SOC (65, 66). The conceptual model on the effects of digital storytelling confirms that storytellers undergo an internal self-reflection exercise and engage in a retrospective sense-making process moderated by the dominant narratives. This process helps storytellers identify their stories’ turning points and transitions, derive meaning from them, and formulate new, more meaningful, and positive narratives (12).

The second analytical theme — The Ripple Effect Of Digital Storytelling — describes storytellers’ collective or social experiences. In the digital storytelling environment, the storytellers find comfort and a safe space to connect and empower each other. The salutogenic model explains that the SOC can be collectively strengthened in a group setting if the group perceives itself as a cohesive unit (67). This is linked to the group´s duration together, size, consensus in their perceptions, and its members’ interwoven self- and social identity (67, 68). Most studies in this meta-synthesis conducted workshops with groups of 6–8 individuals suffering from similar health conditions during three 8-hour long workshops, a level of engagement and group size that makes it plausible for the storytellers to see themselves as a cohesive group. In addition, they appear to achieve a high degree of consensus about their health experiences. Reports confirm that during the group phases, storytellers develop a sense of connection, unity, and community belonging (12, 54). This justifies the assumption that the shared stories and their underlying narratives can also become group resources that storytellers can collectively mobilize and activate to relieve the tension caused by their shared experiences. Thus, it is likely that the digital storytelling process is capable of strengthening the storytellers’ SOC by creating a group setting where each member is empowered to draw on these collective resources (67, 68).

The third analytic theme — Investing And Processing Emotions — denotes that regardless of the narrative characteristics the storytellers are exposed to, creating a digital story evokes a series of affective and emotional responses that can further contribute to a sense of individual and group well-being. Some of these emotional responses reported by recipients of stories can include positive emotions, such as feelings of gratitude or comfort, admiration or being inspired, and negative emotions, such as sadness, distress, or heartbreak (13). Storytellers embrace the opportunity to portray their emotions in a digital story and harness and process them to develop new perspectives of their experiences and move forward (69). Reports indicate that crafting a narrative about health enables individuals to gain a new perspective on their emotional experiences (70) and express and process them through reflection, reframing, or replacement (71). Working on these emotions in this form has been found to have significant health-promoting effects (72), for instance, in people who have cancer by reducing fatigue and improving their general mood (73) or in women with body image and appearance concerns affecting their sleep and eating behaviors by increasing their resilience and reducing physical symptoms (74). It appears that the mechanism behind these benefits involves emotional acceptance, where the storytellers do not seek to suppress or avoid their emotions but accept them, both negative and positive, reducing their emotional distress and enhancing emotional well-being (12). This way, storytellers establish strong emotional bonds with fellow storytellers, fostering unity, cohesion, and belonging. The salutogenic model explains that this type of emotional closeness to a social group contributes to the individual's appraisal that it is worth investing energy and resources to address a particular stressor (30, 31, 75). This appraisal is one of the most critical components of the SOC, meaningfulness, as it refers to the motivational and emotional entity that determines whether an individual is willing to deal with a particular situation (76). Meaningfulness can be maintained or enhanced by investing in four crucial areas of life, including main activity, inner emotions, social connections, and existential concerns (76). The three-phase digital storytelling process acknowledges that people “understand and give meaning to their lives through the stories they tell” (77) and addresses these four crucial areas of life. Thus, whilst it deals with comprehensibility and manageability, it focuses strongly on meaningfulness through reflection.

The fourth analytical theme — Owning The Script — explains that the narrative shift is a deliberate act by the storytellers driven by a newly gained sense of control. The collaborative approach to story construction instills the belief in storytellers that they can steer the narrative in the direction of their choosing. It has been reported that this is a turning point that allows storytellers to re-story their experiences and support their efforts to confront and deal with illness or traumatic events and move on with their lives (78). The salutogenic model can explain this theme by digital storytelling fostering empowerment and reflection that facilitates the identification of the stories and their narratives as resistance resources that influence behavior (62). This is often referred to as gaining personal agency in storytelling, where storytellers actively resist being defined by a dominant discourse and become the active change agents of their own narratives (63, 79). The conceptual model on the effects of digital storytelling cites Bandurás construct of self-efficacy to explain this phenomenon (12). Bandura postulated that self-efficacy primarily emerges through past experiences, vicarious experiences, verbal appraisals from trusted individuals, and emotional arousal (80), all aspects addressed during the storytelling process. According to Fiddian-Green and others, Storytellers work on shared and individual emotional challenges, fostering self-renewal and empowerment while highlighting various experiences that can be acted upon (12). This sense of control also stems from participants’ acquisition of technical and emotional skills that culminate in a digital story (12).

### Limitations

We employed a comprehensive and systematic search strategy but included studies with limited methodological quality. While the reviewers considered these limitations in interpreting the data, recent reports have questioned the rigor of the CASP tool to identify potential methodological strengths and limitations of qualitative evidence (81). Thus, it is uncertain whether using a more comprehensive tool such as the recently published CAMELOT would decrease confidence in the findings. In addition, some of the included studies did not seek to primarily address the experiences of the storytellers, limiting the richness of the data for synthesis. We could not uncover the extent to which each phase of the digital storytelling process provides storytellers with the highest health-promoting value. The data from the included studies treated the entire process as a compound intervention.

## Conclusion

This meta-synthesis addressed exclusively the health-promoting experiences of the storytellers participating in a facilitated, collaborative, and participative group-based digital storytelling workshop. The data provided evidence of moderate confidence that any health-promoting benefits of digital storytelling stem primarily from the storyteller's ability to revise and re-examine their existing stories and the narratives underpinning their lived experiences of health. This process results in a narrative shift likely to contain health-promoting attributes that can shape the storytellers’ health attitudes, values, beliefs, or behaviors. It is clear from the evidence that there is a before and after the workshop. However, considering that both negative and positive narratives could contain health-promoting attributes, there is a need to explore further the types of narratives shared or that emerge in such workshops and provide the strongest health-promoting benefit. Thus, professionals and researchers considering digital storytelling as a strategy in health promotion should carefully monitor the types of narratives shared and the directions that these take. The salutogenic model can explain how the process can be health-promoting for the individual storytellers and them as a cohesive group. Finally, we acknowledge that the analytical themes are not isolated constructs but emerge in response to one another. While we present them sequentially, they do not necessarily represent a linear progression of the storytellers’ experiences. Instead, they interact dynamically, each theme enriching and amplifying the others. This interconnectedness underscores the complexity of the storytellers’ health-promoting experiences and provides a more holistic and nuanced understanding.

## Data Availability

The original contributions presented in this meta-synthesis are included in the article/Supplementary Material, further inquiries can be directed to the corresponding author.
