# Supplementary material for: The health-promoting experiences of storytellers in group-based digital storytelling workshops: a meta-synthesis of qualitative studies
Source: Front Digit Health. 2025 Oct 29;7:1607897. doi: 10.3389/fdgth.2025.1607897 (PMC12605066; doi:10.3389/fdgth.2025.1607897)
Supplement: Supplementary file 1 [file Datasheet1.zip › Appendix 1.docx]

**Appendix 1: Electronic searches**

| **Database: Ovid MEDLINE(R) ALL <1946 to October 30, 2023>**  **Search Strategy:** | | |
| --- | --- | --- |
| #1 | Narration/ | (10283) |
| #2 | biography/ or interview/ or personal narrative/ | (216374) |
| #3 | autobiography/ | (3964) |
| #4 | Analog-Digital Conversion/ | (1660) |
| #5 | Multimedia/ | (2225) |
| #6 | Videotape Recording/ or Video Recording/ or Video-Audio Media/ | (78852) |
| #7 | (video* adj3 record*).ab,ti. | (18543) |
| #8 | (Videotape adj3 Record*).ab,ti. | (849) |
| #9 | (Video-Audio adj3 Media).ab,ti. | (3) |
| #10 | (Analog-Digital adj3 Conversion).ab,ti. | (44) |
| #11 | ((micro or brief or mini) adj3 movie*).ab,ti. | (29) |
| #12 | ((digital* or multimedia or multimodal) adj5 (stories* or story or tales or storytelling or "telling stories" or "story telling" or biography or biograph* or autobiograph* or Narrative* or narration*)).ab,ti. | (555) |
| #13 | 1 or 2 or 3 | (227169) |
| #14 | 4 or 5 or 6 or 7 or 8 or 9 or 10 or 11 | (95841) |
| #15 | 13 and 14 | (385) |
| #16 | 12 or 15 | (920) |
| #17 | "focus groups"/ or "interviews as topic"/ or exp narration/ or observation/ or "qualitative research"/ or "grounded theory"/ or methods/ or "research design"/ or personal narrative/ | (530099) |
| #18 | (("semi-structured" or semistructured or unstructured or informal or "in-depth" or indepth or "face-to-face" or structured or guide) adj5 (interview* or discussion* or questionnaire*)).ab,ti. | (187641) |
| #19 | ("interpretative phenomenological analysis" or "content analysis" or "framework analysis").ab,ti. | (49709) |
| #20 | ((observation* or grounded) adj theor*).ab,ti. | (15451) |
| #21 | ((document* or thematic) adj3 analy*).ab,ti. | (60880) |
| #22 | 17 or 18 or 19 or 20 or 21 | (715912) |
| #23 | 16 and 22 | (347) |

| **Database: Embase <1974 to 30 October 2023>**  **Search Strategy:** | | |
| --- | --- | --- |
| #1 | Narration/ | (17368) |
| #2 | biography/ or interview/ or personal narrative/ | (297567) |
| #3 | autobiography/ | (47665) |
| #4 | Analog-Digital Conversion/ | (2496) |
| #5 | Multimedia/ | (5002) |
| #6 | Videotape Recording/ or Video Recording/ or Video-Audio Media/ | (118466) |
| #7 | (video* adj3 record*).ab,ti. | (25538) |
| #8 | (Videotape adj3 Record*).ab,ti. | (925) |
| #9 | (Video-Audio adj3 Media).ab,ti. | (8) |
| #10 | (Analog-Digital adj3 Conversion).ab,ti. | (46) |
| #11 | ((micro or brief or mini) adj3 movie*).ab,ti. | (36) |
| #12 | ((digital* or multimedia or multimodal) adj5 (stories* or story or tales or storytelling or "telling stories" or "story telling" or biography or biograph* or autobiograph* or Narrative* or narration*)).ab,ti. | (626) |
| #13 | 1 or 2 or 3 | (313482) |
| #14 | 4 or 5 or 6 or 7 or 8 or 9 or 10 or 11 | (135047) |
| #15 | "focus groups"/ or "interviews as topic"/ or exp narration/ or observation/ or "qualitative research"/ or "grounded theory"/ or methods/ or "research design"/ or personal narrative/ | (2758969) |
| #16 | (("semi-structured" or semistructured or unstructured or informal or "in-depth" or indepth or "face-to-face" or structured or guide) adj5 (interview* or discussion* or questionnaire*)).ab,ti. | (237280) |
| #17 | ("interpretative phenomenological analysis" or "content analysis" or "framework analysis").ab,ti. | (56632) |
| #18 | ((observation* or grounded) adj theor*).ab,ti. | (19049) |
| #19 | ((document* or thematic) adj3 analy*).ab,ti. | (74162) |
| #20 | 15 or 16 or 17 or 18 or 19 | (2943088) |
| #21 | 13 and 14 | (4405) |
| #22 | 12 and 21 | (35) |
| #23 | 20 and 22 | (35) |

| **Database: APA PsycInfo <from inception to 2023 Nov 1>**  **Search Strategy:** | | |
| --- | --- | --- |
| S1 | DE "Biography" OR DE "Autobiography" OR DE "Narratives" | (31,473) |
| S2 | TI narration OR AB narration | (2670 |
| S3 | TI ( biograph* or interview* or personal narrative* ) OR AB ( biograph* or interview* or personal narrative* ) | (416614) |
| S4 | S1 OR S2 OR S3 | (439406) |
| S5 | DE "Multimedia" | (3082) |
| S6 | DE "Videotapes" | (2617) |
| S7 | TI Analog-Digital N3 Conversion OR AB Analog-Digital N3 Conversion | (2) |
| S8 | TI videotape N3 recording* OR AB videotape N3 recording* | (271) |
| S9 | DE "Video-Based Interventions" OR DE "Digital Video" | (3064) |
| S10 | TI video N3 recording* OR AB video N3 recording* | (4750) |
| S11 | TI Video-Audio N3 Media OR AB Video-Audio N3 Media | (11) |
| S12 | TI ((micro or brief or mini) N3 movie* ) OR AB ( (micro or brief or mini) N3 movie*) | (35) |
| S13 | S5 OR S6 OR S7 OR S8 OR S9 OR S10 OR S11 OR S12 | (13328) |
| S14 | S4 AND S13 | (2091) |
| S15 | DE "Storytelling" OR DE "Digital Storytelling" | (6141) |
| S16 | TI ((digital* or multimedia or multimodal) N5 (stories* or story or tales or storytelling or "telling stories" or "story telling" or biography or biograph* or autobiograph* or Narrative* or narration*) ) OR AB ((digital* or multimedia or multimodal) N5 (stories* or story or tales or storytelling or "telling stories" or "story telling" or biography or biograph* or autobiograph* or Narrative* or narration*) | (1195) |
| S17 | S15 OR S16 | 6876 |
| S18 | S14 AND S17 | 97 |
| S19 | DE "Focus Group" OR DE "Qualitative Methods" OR DE "Focus Group Interview" OR DE "Group Discussion" OR DE "Interpretative Phenomenological Analysis" OR DE "Interviews" OR DE "Semi-Structured Interview" OR DE "Narrative Analysis" OR DE "Thematic Analysis" OR DE "Observation Methods" OR DE "Participant Observation" OR DE "Grounded Theory" OR DE "Phenomenology" OR DE "Content Analysis" OR DE "Methodology" OR DE "Digital Content Analysis" OR DE "Discourse Analysis" | (113925) |
| S20 | TI ( ("semi-structured" or semistructured or unstructured or informal or "in-depth" or indepth or "face-to-face" or structured or guide) N5 (interview* or discussion* or questionnaire*) ) OR AB ( ("semi-structured" or semistructured or unstructured or informal or "in-depth" or indepth or "face-to-face" or structured or guide) N5 (interview* or discussion* or questionnaire*) ) | (140587) |
| S21 | TI ( "interpretative phenomenological analysis" or "content analysis" or "framework analysis" ) OR AB ( "interpretative phenomenological analysis" or "content analysis" or "framework analysis" ) | (37375) |
| S22 | TI ( ((observation* or grounded) N1 theor*) ) OR AB ( ((observation* or grounded) N1 theor*) | (22572) |
| S20 | S19 OR S20 OR S21 OR S22 | (276143) |
| S21 | S18 AND S23 | (17) |

| **Database: Academic Search Ultimate CINHAL <from inception to 2023 Nov 1>**  **Search Strategy:** | | |
| --- | --- | --- |
| S1 | DE "NARRATION in motion pictures" OR DE "NARRATION in music" OR DE "NARRATIVES" OR DE "NARRATION" | (21472) |
| S2 | DE "AUTOBIOGRAPHY" OR DE "BIOGRAPHICAL films" OR DE "LIFE writing" OR DE "BIOGRAPHY in art" | (18389) |
| S3 | S1 OR S2 | (39,527) |
| S4 | DE "MULTIMEDIA (Art)" | (793) |
| S5 | DE "VIDEOTAPE editing" OR DE "MOTION picture editing" | (474) |
| S6 | TI Analog-Digital N3 Conversion OR AB Analog-Digital N3 Conversion | (45) |
| S7 | TI ( (video* N3 record*) or (Videotape N3 Record) or (Video-Audio N3 Media) ) OR AB ( (video* N3 record*) or (Videotape N3 Record) or (Video-Audio N3 Media) ) | (29,818) |
| S8 | TI ( (micro or brief or mini) N3 movie* ) OR AB ( (micro or brief or mini) N3 movie* ) | (315) |
| S9 | S4 OR S5 OR S6 OR S7 OR S8 | (31420) |
| S10 | S3 AND S9 | (181) |
| S11 | TI ( (digital* or multimedia or multimodal) N5 (stories* or story or tales or storytelling or "telling stories" or "story telling" or biography or biograph* or autobiograph* or Narrative* or narration*) ) OR ( (digital* or multimedia or multimodal) N5 (stories* or story or tales or storytelling or "telling stories" or "story telling" or biography or biograph* or autobiograph* or Narrative* or narration*) ) | (3,284) |
| S12 | DE "STORYTELLING" OR DE "DIGITAL storytelling" OR DE "STORYTELLERS" | (10,690) |
| S13 | S11 OR S12 | (12,730) |
| S14 | S10 OR S13 | (12,906) |
| S15 | DE "QUALITATIVE research" OR DE "CONVERSATION analysis" OR DE "FOCUS groups" OR DE "PHENOMENOGRAPHY" OR DE "QUALITATIVE research in education" OR DE "INTERVIEWING" | (163,667) |
| S16 | DE "SEMI-structured interviews" | (5,971) |
| S17 | TI ( focus groups" or "interviews as topic" or exp narration or observation or "qualitative research" or "grounded theory" or methods or "research design" or personal narrative ) OR AB ( ocus groups" or "interviews as topic" or exp narration or observation or "qualitative research" or "grounded theory" or methods or "research design" or personal narrative ) | (62,163) |
| S18 | TI ( ("semi-structured" or semistructured or unstructured or informal or "in-depth" or indepth or "face-to-face" or structured or guide) N5 (interview* or discussion* or questionnaire*) ) OR AB ( ("semi-structured" or semistructured or unstructured or informal or "in-depth" or indepth or "face-to-face" or structured or guide) N5 (interview* or discussion* or questionnaire*) ) | (176,975) |
| S19 | TI ( "interpretative phenomenological analysis" or "content analysis" or "framework analysis" ) OR AB ( "interpretative phenomenological analysis" or "content analysis" or "framework analysis" ) | (56,753) |
| S20 | TI ( (observation* or grounded) N1 theor*) ) OR AB ( (observation* or grounded) N1 theor*) ) | (21,146) |
| S21 | TI ( (document* or thematic) N3 analy* ) OR AB ( (document* or thematic) N3 analy* ) | (65,361) |
| S22 | S15 OR S16 OR S17 OR S18 OR S19 OR S20 OR S21 | (436,001) |
| S23 | S14 AND S22 | (1,398) |
| S24 | DE "DIGITAL storytelling" | (1001) |
| S25 | S23 AND S24 | (131) |

| **Database: CINHAL <from inception to 2023 Nov 1>**  **Search Strategy:** | | |
| --- | --- | --- |
| S1 | (MH "Narratives") | (20749) |
| S2 | (MH "Biographies+") | (1260) |
| S3 | TI ( biograph* or interview* or personal narrative* ) OR AB ( biograph* or interview* or personal narrative* ) | (278684) |
| S4 | S1 OR S2 OR S3 | (293123) |
| S5 | MH "Multimedia" | (2,253) |
| S6 | TI Analog-Digital N3 Conversion OR AB Analog-Digital N3 Conversion | (5) |
| S7 | TI video* N3 record* OR AB video* N3 record* | (5,992) |
| S8 | TI Videotape N3 Record* OR AB Videotape N3 Record* | (176) |
| S9 | TI Video-Audio N3 Media OR AB Video-Audio N3 Media | (2) |
| S10 | TI ( micro or brief or mini N3 movie* ) OR AB ( micro or brief or mini N3 movie* ) | (95,263) |
| S11 | (MH "Videorecording") | (31,583) |
| S12 | S5 OR S6 OR S7 OR S8 OR S9 OR S10 OR S11 | (130,923) |
| S13 | S4 AND S12 | (9,278) |
| S14 | (MH "Storytelling+") | (5,826) |
| S15 | TI ( (digital* or multimedia or multimodal) N5 (stories* or story or tales or storytelling or "telling stories" or "story telling" or biography or biograph* or autobiograph* or Narrative* or narration*) ) OR AB ( (digital* or multimedia or multimodal) N5 (stories* or story or tales or storytelling or "telling stories" or "story telling" or biography or biograph* or autobiograph* or Narrative* or narration*) ) | (471) |
| S16 | S14 OR S15 | (6,107) |
| S17 | (MH "Qualitative Studies+") | (186,091) |
| S18 | (MH "Focus Groups") OR (MH "Interviews+") OR (MH "Observational Methods+") | (297,279) |
| S19 | TI ( "focus group*" or "interview* or narration or observation* or "qualitative research" or "grounded theory" or methods or "research design" or personal narrative ) OR AB ( "focus group*" or "nterview* or narration or observation* or "qualitative research" or "grounded theory" or methods or "research design" or personal narrative ) | (54,440) |
| S20 | TI ( "interpretative phenomenological analysis" or "content analysis" or "framework analysis" ) OR AB ( "interpretative phenomenological analysis" or "content analysis" or "framework analysis" ) | (36,190) |
| S21 | TI ( (observation* or grounded) N1 theor* ) OR AB ( (observation* or grounded) N1 theor* ) | (14,592) |
| S22 | TI ( (document* or thematic) N3 analy* ) OR AB ( (document* or thematic) N3 analy* ) | (42,546) |
| S23 | TI ( ("semi-structured" or semistructured or unstructured or informal or "in-depth" or indepth or "face-to-face" or structured or guide) N5 (interview* or discussion* or questionnaire*) ) OR AB ( ("semi-structured" or semistructured or unstructured or informal or "in-depth" or indepth or "face-to-face" or structured or guide) N5 (interview* or discussion* or questionnaire*) ) | (115,804) |
| S24 | S17 OR S18 OR S19 OR S20 OR S21 OR S22 OR S23 | (428,065) |
| S25 | S13 AND S16 | (111) |
| S26 | S24 AND S26 | (58) |

| **Database: SocINDEX <from inception to 2023 Nov 6>**  **Search Strategy:** | | |
| --- | --- | --- |
| S1 | DE "BIOGRAPHICAL methods in sociology" | (53) |
| S2 | TI narrati* OR AB narrati* | (49,231) |
| S3 | TI ( biograph* or interview* or personal narrative* ) OR AB ( biograph* or interview* or "personal narrative*" ) | (151,395) |
| S4 | TI Autobiograph* OR AB Autobiograph* | (4,851) |
| S5 | S1 OR S2 OR S3 OR S4 | (192,199) |
| S6 | TI multimedia OR AB multimedia | (1,050) |
| S7 | TI Analog-Digital N3 Conversion OR AB Analog-Digital N3 Conversion | (239) |
| S8 | TI Videotape N3 Record* OR AB Videotape N3 Record* | (40) |
| S9 | DE "SOUND recordings" OR DE "VIDEO recording in social services" | (1,178) |
| S10 | TI video N3 recording* OR AB video N3 recording* | (1,143) |
| S11 | TI Video-Audio N3 Media OR AB Video-Audio N3 Media | (4) |
| S12 | TI ((micro or brief or mini) N3 movie* ) OR AB ( (micro or brief or mini) N3 movie*) | (12) |
| S13 | S6 OR S7 OR S8 OR S9 OR S10 OR S11 OR S12 | (3,428) |
| S14 | DE "DIGITAL storytelling" OR DE "STORYTELLING" | (1,856) |
| S15 | TI ( (digital* or multimedia or multimodal) N5 (stories* or story or tales or storytelling or "telling stories" or "story telling" or biography or biograph* or autobiograph* or Narrative* or narration*) ) OR AB ( (digital* or multimedia or multimodal) N5 (stories* or story or tales or storytelling or "telling stories" or "story telling" or biography or biograph* or autobiograph* or Narrative* or narration*) ) | (392) |
| S16 | S14 OR S15 | (2,119) |
| S17 | DE "CONVERSATION analysis" OR DE "FOCUS groups" OR DE "PARTICIPANT observation" OR DE "CASE method (Teaching)" OR DE "ETHNOLOGY" OR DE "INTERVIEWING" OR DE "PARTICIPANT-researcher relationships" | (50,223) |
| S18 | DE "FOCUS groups" OR DE "INTERVIEWING" | (29,336) |
| S19 | TI ( "focus group*" or "interview* or narration or observation* or "qualitative research" or "grounded theory" or methods or "research design" or personal narrative ) OR AB ( "focus group*" or "nterview* or narration or observation* or "qualitative research" or "grounded theory" or methods or "research design" or personal narrative ) | (22,969) |
| S20 | TI ( ("semi-structured" or semistructured or unstructured or informal or "in-depth" or indepth or "face-to-face" or structured or guide) N5 (interview* or discussion* or questionnaire*) ) OR AB ( ("semi-structured" or semistructured or unstructured or informal or "in-depth" or indepth or "face-to-face" or structured or guide) N5 (interview* or discussion* or questionnaire*) ) | (42,672) |
| S21 | TI ( "interpretative phenomenological analysis" or "content analysis" or "framework analysis" ) OR AB ( "interpretative phenomenological analysis" or "content analysis" or "framework analysis" ) | (12,718) |
| S22 | TI ( (observation* or grounded) N1 theor*) ) OR AB ( (observation* or grounded) N1 theor*) ) | (42,546) |
| S23 | TI ( (document* or thematic) N3 analy* ) OR AB ( (document* or thematic) N3 analy* ) | (12,300) |
| S24 | S17 OR S18 OR S19 OR S20 OR S21 OR S22 OR S23 | (125,298) |
| S25 | S5 AND S13 | (920) |
| S26 | S16 AND S25 | (3,012) |
| S27 | S24 AND S26 | (1,014) |
| S28 | DE "DIGITAL storytelling" | (130) |
| S29 | TI ( digital storytelling or digital story or digital stories or digital story telling or digital tales ) OR AB ( digital storytelling or digital story or digital stories or digital story telling or digital tales ) | (586) |
| S30 | S28 OR S29 | (626) |
| S31 | S27 AND S30 | (52) |

| **Database: Scopus <from inception to 2023 Nov 6>**  **Search Strategy:** | | |
| --- | --- | --- |
| 1 | TITLE-ABS-KEY ( ( analog-digital W/3 conversion ) OR ( videotape W/3 record* ) OR ( video W/3 recording* ) OR ( video-audio W/3 media ) OR ( ( micro OR brief OR mini ) W/3 movie* ) ) | (80210) |
| 2 | TITLE-ABS-KEY ( ( digital* OR multimedia OR multimodal ) W/5 ( stories* OR story OR tales OR storytelling OR "telling stories" OR "story telling" OR biography OR biograph* OR autobiograph* OR narrative* OR narration* ) ) | (7382) |
| 3 | TITLE-ABS-KEY ( "focus group*" OR interview* OR narration OR observation* OR "qualitative research" OR "grounded theory" OR methods OR "research design" OR personal AND narrative ) ) OR ( TITLE-ABS-KEY ( "semi-structured" OR semistructured OR unstructured OR informal OR "in-depth" OR indepth OR "face-to-face" OR structured OR guide ) W/5 ( interview* OR discussion* OR questionnaire* ) ) OR ( TITLE-ABS-KEY ( "interpretative phenomenological analysis" OR "content analysis" OR "framework analysis" ) ) | (610,403) |
| 4 | ( TITLE-ABS-KEY ( ( digital* OR multimedia OR multimodal ) W/5 ( stories* OR story OR tales OR storytelling OR "telling stories" OR "story telling" OR biography OR biograph* OR autobiograph* OR narrative* OR narration* ) ) ) OR ( ( TITLE-ABS-KEY ( ( analog-digital W/3 conversion ) OR ( videotape W/3 record* ) OR ( video W/3 recording* ) OR ( video-audio W/3 media ) OR ( ( micro OR brief OR mini ) W/3 movie* ) ) ) AND ( TITLE-ABS-KEY ( biograph* OR interview* OR personal AND narrative* OR autobiograph* ) ) ) | (7553) |
| 5 | ( TITLE-ABS-KEY ( ( analog-digital W/3 conversion ) OR ( videotape W/3 record* ) OR ( video W/3 recording* ) OR ( video-audio W/3 media ) OR ( ( micro OR brief OR mini ) W/3 movie* ) ) ) AND ( TITLE-ABS-KEY ( digital* OR multimedia OR multimodal ) W/5 ( stories* OR story OR tales OR storytelling OR "telling stories" OR "story telling" OR biography OR biograph* OR autobiograph* OR narrative* OR narration* ) ) AND ( ( TITLE-ABS-KEY ( "focus group*" OR interview* OR narration OR observation* OR "qualitative research" OR "grounded theory" OR methods OR "research design" OR personal AND narrative ) ) OR ( TITLE-ABS-KEY ( "semi-structured" OR semistructured OR unstructured OR informal OR "in-depth" OR indepth OR "face-to-face" OR structured OR guide ) W/5 ( interview* OR discussion* OR questionnaire* ) ) OR ( TITLE-ABS-KEY ( "interpretative phenomenological analysis" OR "content analysis" OR "framework analysis" ) ) ) | (27) |

| **Database: ScieLo <from inception to 2023 Nov 6>**  **Search Strategy:** | | |
| --- | --- | --- |
| #1 | (ti:(((digital* or multimedia OR multimodal) AND (stories* OR story OR tales OR storytelling or"telling stories" OR "story telling" OR biography orbiograph* OR autobiograph* OR narrative* OR narration* or histori* or crónica* or  relato* OR leyenda* OR memorias OR anécdota OR testimonio or cuento)))) OR (ab:(((digital* or multimedia OR multimodal) AND (stories* OR story OR tales OR storytelling or"telling stories" OR "story telling" OR biography orbiograph* OR autobiograph* OR narrative* OR narration* OR histori* OR crónica* OR relato* OR leyenda* OR memorias OR anécdota OR testimonio OR cuento)))) | (2051) |
| #2 | (WoS Subject Categories: Health) (WoS Subject Categories: Medicine) (Type of Literature: Article) | (190) |

| **Database: LIVIVO <from inception to 2023 Nov 6>**  **Search Strategy:** | | |
| --- | --- | --- |
| #1 | TI=(digital storytelling) OR MESH=(digital storytelling) OR KW=(digital storytelling) | (387) |
| #2 | Filter: medicine and health | (16) |

| **Database: BASE <from inception to 2023 Nov 6>**  **Search Strategy:** | | |
| --- | --- | --- |
| #1 | tit:"digital storytelling" tit:or tit:"digital stories" subj:"digital storytelling" subj:or subj:"digital stories" doctype:(12* 13 18*) | (42) |

| **Database: DOAJ <from inception to 2023 Nov 6>**  **Search Strategy:** | | |
| --- | --- | --- |
| #1 | "digital Storytelling" OR "digital stor*" AB | (443) |
| #2 | Filter: Medicine | (45) |
