# Supplementary material for: The health-promoting experiences of storytellers in group-based digital storytelling workshops: a meta-synthesis of qualitative studies
Source: Front Digit Health. 2025 Oct 29;7:1607897. doi: 10.3389/fdgth.2025.1607897 (PMC12605066; doi:10.3389/fdgth.2025.1607897)
Supplement: Supplementary file 1 [file Datasheet1.zip › Appendix 2.docx]

**Appendix 2:**

**Iterations of the Framework agreed by consensus**

**Table 5: Initial Framework developed from the selected two Index studies**

The health-promoting experiences of storytellers participating in group-based digital storytelling workshops (Meta-synthesis, Switzerland, 2024).

| **Theme** | **Community Compassion** | **Deliberate Sense-making** | **Emotional resilience/transformation** | **Gaining agency** | **Moving beyond Vulnerability** | **Shaping or replacing narratives** |
| --- | --- | --- | --- | --- | --- | --- |
| **Description** | This theme encapsulates the core values of social commitment, fostering genuine connections, and nurturing mutual support, which cultivate a collective hope for positive change and growth due to the digital storytelling (DST) process. | This theme reflects how storytellers, through active and deliberate efforts, derive meaning from their experiences and they construct and share their stories. The DST process fosters a conducive environment for retrospective sense-making, enabling individuals to reflect on their past experiences. By looking back, they glean insights that empower them to adopt a more positive perspective on the future through prospective sense-making. | The theme captures the storytellers' process during the DST to develop emotional resilience. Through exploring and understanding their experiences, storytellers embark on a path of acceptance, releasing emotional burdens, adapting to challenges, and persevering through difficult emotional terrain. Ultimately, this journey leads to personal growth and development, reflecting the transformative power of storytelling in nurturing resilience. | The theme captures how storytellers develop a sense of control over their own narratives. The DST allows for a process of self-discovery in which the storytellers are empowered to tell the truth  about their situations, sometimes to produce a counternarrative or to send a message to others. They build confidence through their fellow support and a sense of empowerment. | This theme embodies the poignant journey of storytellers as they transition from vulnerability to comfort. They often grapple with the impulse to conceal their narratives out of fear or self-protection. Despite the initial apprehension and reservations, they courageously unveil their truths, fostering empathy and forging deep social connections with fellow storytellers. | This theme encapsulates the transformative power of digital storytelling in reshaping individuals' understanding and portrayal of their experiences and events. Through this process, participants often undergo a profound shift in perspective, leading to the emergence of new narratives by altering, reshaping, or even replacing their old narratives altogether. |
| **Codes** | Being cared for | Dedicated engagement | Authentic experience | Accepting all narratives | Comfort Transition | Counternarratives |
|  | Being Heard | Future Orientations | Cathartic | Active participation | Embodied Shame | Narrative negotiation |
|  | Camaraderie | Impact beyond workshop | Emotional experience | Confidence building | Initial Concealment | Narrative Transformation |
|  | Community Bonding | Prospective sense-making | Emotional healing | Raising awareness | Shaming potential | Narrative resonance |
|  | Empowered by others | Retrospective sense-making | Emotional release | Social acknowledgement | Self-protection | Positive Narrative |
|  | Feeling valued | Seeking Closure | Emotional work |  |  | Pre-existing narratives |
|  | Hope in the collective | Self-actualization | Persevering |  |  | Telling truth to others |
|  | Social commitment | Self-expression | Relief |  |  | Narrative liberation |
|  | Solidarity | Sense-making |  |  |  |  |
|  | Supporting others |  |  |  |  |  |
|  |  |  |  |  |  |  |

**Table 6: Mapped and Organized Study Quotations Within the Framework**

The health-promoting experiences of storytellers participating in group-based digital storytelling workshops (Meta-synthesis, Switzerland, 2024).

| **Study** | **Community compassion** | **Deliberate Sense making** | **Emotional resilience** | **Gaining agency** | **Sense of Vulnerability** | **Shaping or replacing narratives** |
| --- | --- | --- | --- | --- | --- | --- |
| **Beltran and Begun. (2014)** | I guess what I want to do now is help. (the community, the maori)  This reclamation also provides a framework for fostering empathy and extending grace when considering the manifestation of social problems.   I really enjoyed it. And then I thought, well, they’re going through the same process I’m going through. So they must be feeling a little bit of what I’m feeling.  I’d like to do a workshop…Like anything, when somebody’s suffering at like, say, if you’re boozing your heart out, and if they saw that story…’cause it can only be told from somebody in…that’s their truth… they’d connect immediately to it, because people are liberated when you… oh, Nelson Mandela said it in a speech: ‘You liberate others when you share your truth.’ (Moana)  I definitely want to take my story and show it to my iwi, my family, and I want to see if they would be interested in doing some work of this nature. I feel like I’ve been given something that will help me help someone in a very simple way… | We obviously had a history of [HT], but we didn’t have anything to describe it…I’m conscious of it now. It’s like, ‘Oh yeah, is that what that was? It just was something we never though about, but now it makes sense.’ (Moana)  So I think that helped me realize. [HT had a huge impact on my life. Like a huge impact on my life. I could be a native Māori speaker right now if it hadn’t happened.’ (Tui)  Learning about HT] was a bit of an eye-opener for me, because it was like, ‘True…’ And I think the workshop gave me an appreciation for it.  I know we’re all at different stages, and being inheritors of that trauma, we all deal with it at different levels in different ways, but I get it now.  [Learning about HT] was a bit of an eye-opener for me, because it was like, ‘True…’ And I think the workshop gave me an appreciation for it. I still see the historical trauma in my family.  it was kind of like a powwow, where we all came together and really understood where each other was coming from. (Tui) | And I’m a bit more forgiving of my iwi than I probably was, and maybe a bit more forgiving of myself.  I grew. I was liberated and at peace. I’m at peace with myself in a way that I’ve not been for…I don’t know…probably forever…by getting [the story] out, because I harbored it for so long. That’s not a healthy way to be. It really makes…well, talking about health and trauma… it is medicine. That’s the best way I can say it. (Moana)  Making [a DS] was definitely empowering. Empowering in the sense that it’s actually released that pain that I was holding on to…it let me let go of it. It helped me to let go of it, the pain of it. (Aroha)  I just feel so cleansed by the process [of DS]. I can’t tell you how large I feel…it’s made me a better employee, because I’m at peace with myself in a way I wasn’t before through this process. (Moana)  [It was] definitely significant when I actually read my script to everybody else. I can’t describe that. It was just… really empowering. It was really a special moment… for someone else to hear what I’ve been thinking and feeling; and just to put it out there was incredible.  So it made me feel safe in that environment for all of us to be in the same boat, in the same position, to bring forward a story. And then when everyone read their story…it was so powerful hearing everyone’s story. It was like, ‘Wow.’ I related to every single story. It was like, ‘Okay. It could be one of my stories. Wow…we really are connected.’  And it’s healing. It’s healing to know that you’re not the only one that’s been through that pain. You’re not the only one that has felt lost in your own land, and you’re not the only one who has suffered…suffered growing up trying to find your identity. So, yeah, that was really…that was healing, speaking it out. That was healing, hearing other people’s stories. (Aroha) | You lose your knowledge base and you lose your community base and you lose your identity. It’s some pretty fundamental pillars…you knock the pillars away, you break the base. (Whetu) |  | It’s made a presence in my life in a way that wasn’t [there] before. [It was] quite powerful . Liberating..  These interactions pave the way for an important reframing process towards healing, as by better understanding past traumas and the many resulting and ongoing toxic by-products, this deeper understanding emphasises healing and the reclamation of cultural identities and values.  Life-changing stuff. You can see it. You can hear it in people’s narratives. It’s about talking through their story. You can hear the growth and the de-cluttering. Yeah, it’s almost like watching a flower, like a peony rose… beautiful flowers…and it’s just like watching them burst into life.  It just changed my whole perspective on everything. And I think being in those places and hearing other people’s stories as well, it does make you more proud. |
| **Boydell et al. (2018)** | Young people appreciated sharing their  experiences with others who had similar stories that they could “vent to” and “who won’t judge you.” Taylor shared that she was now less afraid to open up about mental illness with others and that she would write more songs  about her experiences. | t was “pretty cool making the story as I got to learn more about myself.” | They identified the positive impact creating their stories had on them and saw the potential effect it could have on others. Both Anna and Luke described the freedom of being allowed to “open up,” |  | For instance, although creating the story was emotionally difficult and “scary” for participants, they reported feeling better and a sense of relief when it was completed. | “I’m trying to think of the word but it was just like, just like opening up and everything, just made you feel good. I just learned that the videos can have a really big impact.  Elyzia stated that creating a video enabled her to put her past behind her. She now felt that she could be productive with her ideas as she learned how to write and tell stories and realized that she is worthy.  “It feels good knowing that like even though, like, I went through shit it’s going to help someone else, you know like even though I suffered it will help someone. That feels good.” |
| **Briant et al. (2016)** | The digital storytelling process had great power for the participants. It created a sense of community by allowing people to share their stories and connect with others around their experiences.  “We’re also here with the same disease that anybody can go through.” Another participant shared, “I thought it was fantastic. The meeting of other cancer patients. Their stories. We all had similarities. We’ve all been through the journey that you don’t wish upon anybody.”  “When I was diagnosed, I would have liked to know something more about this illness…it was difficult for me because I didn’t have any information…making this digital story, I feel that other people can have at least a base or an idea of what one goes through.” | Quotation:“…there were tears in my heart and in my eyes…because finally I learned what it meant to live with a chronic disease and how difficult it was…it helped me so much to create a digital story.” | “…it felt good to talk about the topic.” | “…this digital recording it’s very important because we’re making it known…we are letting our community know that many things can be prevented.”  “I think it can help because one learns from listening to other people and telling a little bit of our story as one that has lived the illness, how did we survive, how have we been, what have we done to feel better? I think that it helps other people if they are going through the same evil to trust a little of that to see if it works from them too.” | “Well, when I got the invitation, I said, “Can I really make a digital story? No, I don’t qualify, right?” I said, “No, this has to be for someone very extraordinary.” Another participant said, “We didn’t think that we would be suitable for many people to see us on the Internet.”  One participant said that being put in front of a computer caused them to “momentarily freeze.” | “It did surprise me [to see] the story once I finished it. It was something different for me, seeing it from living it.” |
| **de Vecchi et al. (2017)** | .. . I think it can allow someone to enter that space with you and someone might not have that lived experience, but that story you’re telling might map onto some other experiences they’ve had ... you can create a bit of shared space and shared understanding ... and the contextual stuff is key ... I think the use of imagery and music .. . can tell a story ... that words can’t ... | Participants described digital storytelling as a creative process that enabled them to understand their own and others’ beliefs, perspectives, and life experiences.  When we all got together and shared experiences, that was really moving and a real eye opener to see what other people had been through, and other people’s perspectives on wellness and mental health.  Some participants identified personal qualities previously unrecognized.  . . it makes something complicated easy ... To make those layers like that .. . with all the options of using multimedia .. . I thought it was a very useful tool to express my journey.  I think that art is a place where people can make meaning and it’s a way to be a balm for suffering. | .. . I found it challenging because some of the stories wer-  e .. . emotionally resonating ... you felt for what people go through, what’s behind that outer shell that you show to the world. | One of the things about here is that there are no masks, that mask has gone, you are who you are .. . They dissolve. It doesn’t really matter, we are just ... ordinary people who have an interest in learning, but at the same time trust, it takes a trust in each one of us, and it works. | Support was seen as essential for overcoming fears, developing confidence, and learning through exploring and playing with the digital medium and story  At first, I was afraid of it cause I’m afraid of technology ... but I was comfortable with it towards the end, I think that fear comes out of the unknown, so not knowing what you’re doing ... once someone showed me, I picked it up relatively quickly.  I didn’t feel like anyone was going to physically or emotionally harm me ... Groups intimidate me ... I’ve had bad experiences in groups before .. . I think the group treated everybody else with respect. And they were all equal. There was no “I’m a clinician and you’re a consumer,” and therefore you’re either more important or less important.  Issues with the fact that th facilitator has a powerful position and how to mitigate this. This is beyond what other say. It may be a contradictory finding. Explore. |  |
| **Difulvio et al. (2016)** | the elicitation of feelings of empathy from both telling and listening to stories, and the importance of being listened to when telling stories, all of which were connected to the development of social support and social ties.  Being listened to and in turn listening to others was akin to  feeling valued and cared for.  “I really felt like the people in this workshop really cared about us girls. It feels good to know someone cares.  “like I was really listened to.”  I know I ain’t alone;”  “it was a very deep impact on me because people go through diﬀerent things even though we are mothers;” “I’ve realized everyone has something they want to share but don’t know how;” “I felt like it could happen to me or to people that live around me. Some people have the same problems.” |  | realizing catharsis from telling a story,  “[I] feel no more guilt now that my story is right,” and others who spoke of the value of “open[ing] up” and “let[ting] go” of “things we’ve kept inside.”  “it felt good to be a part of this workshop—you learn more about yourself and other people.” | “it’s more important to say your own story than someone else [telling it] because it’s the only truth, cause it’s told by you.” Another wrote, “[my story] was FACTS, not assumptions. It comes from the heart instead of someone maybe trying to hurt us.” A third participant agreed: “it was a good way for all of us to get to tell a story and get real information [instead of] someone else telling false information about our story.”   feeling “conﬁdent,” “strong and respected,” “optimistic,” “accomplished,” “awesome!” and proud of “speaking up [and] letting my voice be heard.”  She felt a “motherlike strength” as a result of sharing her story with others. | catharsis was accompanied by anxiety and nervousness from sharing “with people I don’t know” and for another participant, “embarrass[ment from] opening up to new people.”  the two participants who recalled initial anxieties also reported feeling proud of being able to speak openly about their stories in front of the group. | The young women wrote about being proud of “actually making the video,” “that I ﬁnished on time and that  came out amazingly,” and “actually attending the workshop and being here every day on time.” |
| **Dixon & Isaac (2023)** |  |  |  | After both authors viewed and debriefed their final digital stories with each other, the second author expressed how important the DST process was in her healing journey:  People refuse to acknowledge our experiences and our beauty, this space allowed me to do that. I can be loved, I can be accepted. |  |  |
| **7 : Ferrari et al. (2015)** | Participants described how the group setting helped them to ﬁnd comfort, support, and reassurance that they were not alone in their journeys and emotions: “It reminded me that I wasn’t alone. Not only are there others who share my experiences, but [they] share my view of the experiences. | Every time I watch it, I take away a greater understanding of why it happened to me.”  At the same time, digital storytelling harnesses the use of creative media to enable people to access multiple ways of conveying meaning in order to represent their experiences. | According to several participants, the storytelling process provided an opportunity for releasing emotionally fraught memories and stress-ﬁlled experiences. Participants described their storytelling as cathartic: “Not only did I open up to the group,  556 ps.psychiatryonline.org  but I opened up to myself.  “[I told my story] many times, until I got tired of the situation and became apathetic to it. It started off as deeply personal, and deeply emotional, but I later stopped telling the story because it became a mundane, everyday thing to have my brother act this way.” | It felt like Iwas taking a level ofownership over the story that I hadn’t felt before.Itfeltreally goodto ﬁnish it—because I work in the ﬁeld [and] experiences rarely feelliketheyhaveaclearend point—this one did, which was very refreshing.” |  | This experience is one that will carry on with me forever. If anyone asks me [about] my condition, I now have a perfect package to relay what has happened.  Every time I watch it, I take away a greater understanding of why it happened to me.” |
| **8 : Fiddian-Green et al. (2017)** |  |  |  |  | “I Hadn’t Planned to Tell”: Dalia’s Story  However, as the workshop progressed she became more quiet and withdrawn, removing herself from the group at large. Up until the last moment, she refused to share her completed digital story, despite sharing her story with the group during the story circle on day 1 of the workshop.  In order to depict the sense of imprisonment from the silences that she must maintain in order to “fit in” and not “be an outcast,  Monica was viscerally quiet and shy during group work throughout the workshop. |  |
| **9 : Goodman .(2018)** | “A lot of people down here, they get in this rut, you know, and it just seems like there’s no way out. I want to say that there is a way out and it can be very rewarding.”75 | She stated she wanted to make a connection between her suffering and her heroin use and communicate this information to the viewer. She said: “Because it [her grandmother’s death] . . . set me on my self-destructive path. It’s what caused that sense of loss and like I didn’t belong anywhere.”70  Her response suggests that Marie deliberately tried to produce a counternarrative by focusing on her inner experiences and not on the act of consuming an illicit substance. | Marie’s willingness to examine an emotionally challenging moment in her life and describe how it impacted her is compelling and courageous.  Marie’s willingness to share these deeper and personal elements of her life seemed to allow her to bolster her self-conﬁdence. In her postworkshop interview, she stated: “It kind of makes me want to feel better about myself. It makes me want to not be so hard on myself.”  I guess it’s just perseverance, you know. | Marie’s digital story is unique in the way that she highlights her sense of self-worth, inner strengths, and determination to carry on.  “It’s real. It’s true. It’s to the point.”70 She added that viewers are able to “see the story behind me” and not the “actual drug use.”  My story is real and truthful. I think that sometimes the media doesn’t have a real story, you know? That they’re just putting it together from bits and bobs they’ve collected from different people . . . I told the real story. It came from my heart . . . . Real means to me just honesty, very matter of fact—all facts from my point of view. And I have experience. I’ve been down here for 30 years. You know I’ve seen a lot and did a lot. I don’t think that anyone can know what that is like until they actually go through it themselves. So it’s hard to put together a real story unless you’ve been there and done that |  | Digital stories such as Marie’s could also inform the  public and policy makers about some of the positive qualities of chronic and vulnerable heroin users.  “It’s real. It’s true. It’s to the point.”70 She added that viewers are able to “see the story behind me” and not the “actual drug use.”  I guess it’s just perseverance, you know. I know that my grandmother would want me to be here still. She would want me to get to enjoy the beauty of life, to see things that she has seen and not suffer. I’ve suffered long enough. It’s time to enjoy life. It’s time to move on and not be sad and lonely like I have been.69  “I’ve set some realistic goals that are attainable.”71  I wanted to get across the point, being, you could have something detrimental happen to you, you can either take one or two roads, I took a road that was close to me dying but through people helping me and getting me the right support I managed to get my life on track . . . I think for myself it was an effective way in telling my story because a lot of people can relate to having something happen detrimental in their life.74  “I wanted to clean up my life. I wanted to live again.”75  People tend to think that drug addicts are all bad but we’re not, and I think that people need to know that, because I think they forget. When you’re in this life for so long, you forget how to feel good about yourself. And, I don’t know, I think people need to know that just because they’re using or if they fall off the wagon, you can still get back on it, and keep going and it’s all good.76 |
| **Gubrium et al. (2016) INDEX** |  | Participants spoke of the immense relief they felt from telling their truths to others about challenging experiences.  It’s like, it’s a story, but it’s pretty much pointing everything to my best friend and I kind of felt bad . . . So seeing how I could turn it around into an actual story, where I’m not really pointing fingers at anyone? It was like an eye opener. I felt like I wasn’t as ignorant as I was before.  Being listened to, and in turn listening to others, evoked feelings of being heard and acknowledged, something that most participants note— either consciously or unconsciously—as typically lacking in their lives.  or unconsciously—as typically lacking in their lives. Being heard nurtured a sense of being cared for by a community. Participants commented on the relatability of each other’s stories, while also expressing moments of revelation when realizing that their stories are rarely heard.  . A tap on the shoulder, a smile, a nod, an affirmation all confirmed that other group members listened to and understood the stories, outwardly signaling a commensurability of experience to build a sense of solidarity  , Paola related that the workshop was fun, allowing her to be a part of a supportive group of “new friends.  . . . it was nice to see [my digital story up on the screen], other people, like, actually paying attention to me. It made me feel, like um, that someone was actually listening to my story. ‘Cause when I tell people, it’s just like, “Okay, whatever, your life’s not over, that’s great.”  Nicole taps her on the arm and smiles at her.”  I felt like I was supported, I felt like I pushed through it . . . pushed through being scared and . . . it helps. When there’s  people who really care around you . . . it shows me that there’s people out there who care even if they don’t know you | “getting it off  my chest,” “letting it out,” or “seeking closure,”  And, it’s like, making us break our shell, because we had a couple people that like, you know, told a rough story, and it’s not only a relief for yourself, but it’s like, giving a message to other people that, you know, you’re not alone.  I felt like I was supported, I felt like I pushed through it . . . pushed through being scared and . . . it helps. When there’s  people who really care around you . . . it shows me that there’s people out there who care even if they don’t know you | DST process as helping to break the protective “shell” of untruths they have told about themselves and others, giving them the courage to bring to light their own lived experiences.  We are telling our story, but we’re also learning that there are people out there that, like, do need to know. | I think I gotta start telling the truth about me and my son, what’s going on. ‘Cause I just be like, “Lie to people about your problem” . . . [but] it’s not really gonna get you nowhere. If you just lie to people about what’s going on, they’re not really gonna help you. In ways that you need help. So that’s why I was like, “alright, I’m just gonna have to just be honest and just . . . tell them what’s going on.”  Gubrium also wondered if some of the pregnant or parenting participants in the group would feel shamed by some of the other participants [nulliparous group members’] comments on avoiding the “mistakes” their mothers had made by “doing things differently” and not getting pregnant at a young age. | peeling back layers of untruths that they had previously felt compelled to tell to protect themselves and their families from the judgment of others within and outside their community.  The DST workshop afforded participants the time and space to create stories that “talked back” through truth telling (hooks, 1989; Spivak, 1985) and move beyond a discourse of shame. |
| **Gubrium et al. (2019)** | During her follow-up interview, Dawn explained that she used her digital story as a device to communicate with her mother and grandmother about what had happened. She had connected with her mother after learning that she too was a survivor of sexual abuse as a child.  The sense of solidarity developed from working together with a group of fellow pregnant and parenting young women from the same community (in workshop three) was another step, allowing Amara to, as she put it, ‘face her past demons’.  Amara began quietly, prefacing the reading of her script by saying that her story focused on ‘learning how to have sex that wasn’t scary, where something wasn’t done to her’. Fellow group member Flor responded, ‘Can I hug you, is that a possibility?’ Natasha and Pilar both chimed in that it felt ‘nice to know’ that they were ‘not alone’. The story circle continued, with other participants telling their stories of trauma and fellow group members providing supportive commentary throughout |  |  | They thus transformed experiences that had previously objectified them into an object of their own representation, which afforded them a sense of agency (and in our evaluation of outcomes, an increased sense of social support, self-efficacy and self-esteem).  Amara said that her message was to encourage others – including fellow group members – to speak up and openly about these experiences and not allow what she sensed as local norms on stigma and shame around sexual violence to silence them.  Paola, another group member, concluded at the end of the story circle that it is ‘brave of us to share our stories – it teaches us as mothers, parents – that these are the things we can teach our children – about domestic violence, birth control. They don’t have to go through the same thing. This brought us together as mothers and people’. | the DST process provided a safe space for participants to open up about what were for many participants previously stigmatized and shaming experiences with violence and trauma, while encouraging them to reconfigure themselves in the situation |  |
| **Howard et al. (2023) INDEX** | All participants indicated that they were feeling quite exhausted from the week, and I am astounded that they all still showed up for the workshop...Even more astounding was that they had all prepared a story to share.  The process of connecting to share common life challenges beyond endometriosis appeared to be unifying and affirming.  Sharing this experience with other storytellers with whom they could relate and the nature of the facilitated story circles resulted in immediate camaraderie. The connections they built with each other throughout the sessions allowed them to connect as people, not just as people with endometriosis, as a storyteller shared:  I was very moved by [name] story as so much of my own is captured in her words. Again, I felt a sense of connection and of a deep understanding from my fellow participants, which was really beautiful and made me want to think about how to connect further with these women or others who have Endo  They conveyed hope in the collective, with a storyteller commenting on the “many shared experiences uniting all of us.”  All storytellers (6/6, 100%) characterized the workshop as a supportive group and a safe, respectful space wherein they felt cared for. Support was offered by all storytellers in all sessions in the form of words of gratitude and encouragement when storytellers shared their experiences or stories and acknowledgment and acceptance of the emotions that arose. The following phrases were offered to fellow storytellers after they shared a draft of their story script: “beautiful,” “incredibly powerful and deeply vulnerable,” “writing is exquisite,” “speaking so openly,” and “I can relate to what you described.” Furthermore, as the workshop progressed, the connection with coparticipants grew into a sense of belonging, camaraderie, and community and a desire to continue supporting each other and others affected by endometriosis.  Actually, hearing that others genuinely have the same experiences as me. This session was sad and devastating. It was hard to hear how much Endo and pelvic pain had negatively affected their lives. | For me, it has been a journey to get to know myself better...how I want it to be perfect, or wanted to show many aspects, and I have to let it go. [  What this video did for me was helped me to recognize that I have been doing so much work on listening to my body and what my body is actually telling me. And sometimes, I get a little left-brain where I’m over-analyzing and thinking. And so, when I pull myself back into my body, there’s a pretty clear message that like, [something] just doesn’t feel right. | a complex emotional experience  I had a very emotional week with getting the video together as it took me back to that time...My story just made me really sad that I now will never be able to have my own kids even though I had decided to make that decision years ago. I haven’t felt myself be this emotional about not being a mom, about how my life has changed, for a few years now. I felt I had been doing well up until a few months ago, but this stirred up old emotions which I thought I had moved past and was ok with. But I think when you wanted something so bad, you accept it didn’t happen, but it can still hurt.  The sharing of such personal and private stories was particularly emotional, evoking feelings of sadness, anger, and grief, which came as a surprise and was described as hitting some storytellers harder than they had expected.  Hearing and viewing the stories shared by the other storytellers also evoked strong emotions of sadness, anger, and grief.  “man, am I ever wiped out!”  Although some memories were emotionally painful, this work of vulnerability and revisiting losses seemed healing rather than inciting reinjury  The storytellers reflected on the emotional but ultimately healing process of revisiting their own stories, reading their scripts aloud, hearing their voices, and seeing the finished product:  I felt when reading the script itself, it took me back to that time, and I couldn’t believe what I went through. I was also having a rough week with my external world and comments being made about how life isn’t life if you don’t have kids. This, of course, made me emotional, so I thought reading and revising my script would be difficult, but instead of feeling weak and unworthy, I felt strong and brave thinking of all I have overcome.  Affirmation and validation from being believed by fellow storytellers, as well as empathy and connection with others’ stories, were prevailing emotions. | Bearing witness to vulnerable emotions seemed to ignite unity and a normative drive to tell it like it really is.  The storytellers’ commentaries conveyed their sense of pride from persevering through the project despite some difficulties and accomplishing a personally meaningful goal, as evident in the following journal entry: | In the beginning, I was really, really anxious about it, but even halfway through the first session, that anxiety definitely decreased just at how welcoming and accepting and just how common it felt our experience was. We had this like connection that was beyond just Endo, having Endo. It was really great.  included intense  vulnerability stemming from excitement and commitment to the research but coupled with fear and anxiety  The storytellers expressed fear, anxiety, and worry about who else would be in the workshop before it began but also because they were asked to reveal personal and private details.  It definitely felt raw and vulnerable, of course, to talk about the painful sex aspect and simultaneously felt super important because this is such a stigmatized  aspect of endo that I think is just so important to talk about and to raise awareness over  So much of our lives are open to people, to doctors, to nurses like how many people have seen me naked. It’s ridiculous! So sharing my personal story like this, it doesn’t really feel that personal anymore...to be encouraged and have a platform like this...we want you to be honest. It was very refreshing. | Even though all our experiences with Endo are nuanced, there are many common themes—validation with diagnosis, importance of support networks, feelings of loss, hope even amidst great struggle. We are stronger than we may sometimes feel. |
| **Jun et al. (2022)** | a sense of increased human connection through shared stories; realizing they shared more in common than they had realized allowed them to better relate to their peers. As participant 4 explained, “I find myself relating to every single story. I have a story within that story … and then I think maybe I need to get to know someone else.” This sentiment was shared by another participant 7, “Listening to [others’] stories … feels so much a part of something bigger than my own personal experiences.” | Nurses are always there to pick up the pieces for everybody else. We have our stories, but we have our own trauma; we have our own issues. I think what the storytelling does is dig into nurses and [expose] some things we don’t want to disclose. We put all that in a backburner [sic] because we have to do everything for everybody else. | Maybe it’s the shared emotions like sometimes the release of the emotions …. Being heard and being seen as a human, not just as a nurse … Our whole job is all about secondary trauma; hearing stories that are so challenging that you have to be able to put that somewhere … storytelling is a way to do it. I felt significantly more energy afterward and the ability to be kind to my client. |  | I think there might have been one person that wasn’t quite ready, maybe being vulnerable. But it might be a little bit easier if they know that other people on their team are participating. Being around complete strangers for three days is kind of terrifying. |  |
| **Kim et al (2020)** | Workshop participants frequently verbalized experiences of building community and a sense ofsolidarity through collectively sharing, listening, and engaging with each other’s experience in a supportive group environment.  I could totally relate to your story. As a mom, I totally feel your emotion.  I think it was great when everyone talked and supported each other. Number one, you heard their story you understood where they were coming from because you can actually feel it and connected to those who experienced the same thing.  I felt great that someone who who went through the same experience was listening to my story with a full ofemotion and support instead of getting advice from somebody who did not experience it.  Specifically, participants engaged in the social construction of caring by transforming what it means to give care and to get care during the workshop. This interplay between the caring and the cared-for illuminates the healing effects of community and self-identity in relationship to another.  One ofthe nice things I need to say is get to know these individuals. We have been here for 3 days together. And now I feel like we became good friends. We will form our own support group in the future.  Because you wonder if it’s ever going to end. I mean you wonder if it is ever going to get better. I know that I would ask you and (name), does this ever get better? Is it, and they would be like yes, yes it’s just you know. I don’t know why I have so much faith in these guys.  You know what I think it was so therapeutic for me was thinking that I was helping somebody else. Somebody else will see this video. So it wasn’t necessarily that I got to talk to other people. I was telling my husband that I’m going to help others, I’m doing it to help somebody. |  | It was so nice to hear from other patients who have gone through what you’ve gone through and what other people have gone through. It was more than talking and having a support group; it was very therapeutic for me.  I was surprised at how much emotional I was when I went over my real story. I never thought ofonce any ofthe feelings that I was having. This workshop opens me up to realize the value of talking and sharing emotions. Now I am sharing my emotions with my family. | That’s a very brave thing to talk about. So, I don’t want you to get scared. I felt that that is a very powerful story and I think you are right person to tell your truth. You can do this and we can do this together.  I usually don’t talk about my emotions because I don’t want my family to worry about me. It took me a while but I felt so relieved. This is therapeutic. It was an amazing, sweet, pleasant experience so thank you very much.  Self-empowerment Participants said they gained a sense of achievement and self-empowerment through the creation of their stories. Specifically, participants expressed that observing other people sharing their own story gave them confidence that they could do the same.  Your story really touched me and inspired me. I was a bit afraid of sharing my story in a group, but I think I can do that now.  I’m pretty proud of mine. I really felt good about myself that I did everything. |  |  |
| **Kim et al. (2023)** | I was so compassionate, and they made me felt for her [another study participant] We are all moms, and we'll have it all figured out and I guess we learn along the way, and that's really great. |  |  |  | . Although sharing one’s story in a group could be stressful, they found it rewarding. |  |
| **Laing et al. (2017)** |  | Cause it’s difficult to express how you felt in a moment just off the top of your head. You need to spend some time with it, you need to sit with it and you need to write your story and formulate your sentences and craft your words, you know?  Like in the last 3 days, every time I watch it, it . . . it’s like you don’t give yourself enough credit for going through that much and just watching that . . . oh wow, that’s actually amazing (laughs). I never thought of it like that, but when you put it all together, that’s . . . that’s really awesome.  So it wasn’t easy, to share my story, but I guess it’s being able to step back from those immediate moments, and look at them a little more objectively. It really helps to make senseof things, make meaning, and see how your story is connected to other pieces of your life. (Mark)  I needed to make sense of it [the experience of having cancer]. And I think just because—I think in particular the millennial generation—we’re looking for meaning, and I just wanted to find some meaning in what I’d been going through. (Mark)  I didn’t—couldn’t—make sense of it while I was in the thick of it [treatment for cancer]. It wasn’t until later that I realized I had so much shit I had to deal with—psychological shit, you know. I guess until it’s dealt with, it’s not dealt with. | I think with digital stories you’re just way more thoughtful . . . the images and the music allow for more of the emotion to come across . . . they allow you to feel things.  I don’t know. I really don’t know. (thinking) All of those hard feelings . . . the hard feelings and the loneliness I went through. That stuff is all off my shoulders now.  It’s a side of me that a lot of people didn’t know about or didn’t see, even among my close friends I think there are those that don’t know about this. People have their secrets. And um . . . it’s been kind of cool opening up this part of my life to them because they see a different side of me . . . and just kinda like . . . the story of who I am. I mean, I almost started putting less emphasis on it as a problem in my life [after making a digital story] because it was a secret and it shouldn’t be problem anymore because the secret is not a big deal. (Ian)  I didn’t think it [making a digital story] was going to mean anything profound because I just thought “Oh yeah, I have shared my story, I can help share it for whoever you might show it to at the hospital,” but I didn’t realize how healing it was going to be for me. (Cate)  I think about the weight off my shoulders as being the best thing about making a digital story.  “therapeutic, not therapy”  The making of the video was out of anger, like you know, I just wanted to get it out so bad. And yeah, it’s a little hard to explain. . . . Like I just let it all spill out on the video, kind of thing . . . I was really angry when I was looking at those pictures. (Olivia) | Most of the participants in this study told us that making their digital story was a way for others to understand their experience of cancer.  I think it [my experience] just had to be known.  This desire to have others understand “what it was like” was repeated over and over in the interviews.  My goal was to make the person feel uneasy with it, with the whole thing. Each image I put in there was in there for a specific reason. Every picture I had was representative of what I was saying in my story. (Ben)  I was feeling so lost . . . and then now I just kind of feel filled with so much purpose. Just being able to share your story with someone who wants to listen is . . . well . . . I, I think we often don’t realize that something simple and little like that can be so helpful. (Anna)  Telling my story and kinda expressing it in this way, helped me heal a little bit more, than um . . . than I have in the past. I didn’t worry about what other people may think of it . . . what pictures my mom would or wouldn’t want me using. It was more about finding truth in some of this things. ( | The more you tell your story the easier it gets . . . the easier it gets and the less scared you are talking about it. (Mark) | Digital stories thus contribute to the sharing of experience, helping people to speak differently and to hear more inclusively  They can put the maker of the story in a place where they can understand their experience of cancer differently and the viewer of the story in a place where they can better understand the experience of the person with cancer.  a brick in my pathway to healing.  It gives you something to do . . . a little more than doing nothing. It gives you something else to think about instead of just lying in your bed or whatever.  I feel a lot different [after making her digital story]. Cause I’d kept it up inside for so long that to finally let it go was a weight off my shoulders. Like, it was really a relief.  I think it was making the video and putting the pictures in, it was kinda like . . . I got through it [cancer]. I don’t think it’s that bad anymore, to look at those pictures. I think I’ve just gotten used to it now. I hadn’t looked at those pictures since—like before I did the video, I hadn’t seen those pictures, so it was just a bit of a shock to see. When you’re healthy and you look at those pictures and you’re super pale and bald and sick (laughs). But I’m none of those things anymore. I got through it. (Georgia) |
| **Laing et al. (2019)** | I think sometimes, when you think back about what you were thinking or what you were feeling at a time that was so horrible [referring to cancer treatment], it kind of lets loose some of the stress that has a hold on you, I guess. And I think that just kind of verbalizing it, and making this digital story, and knowing that somebody may see this, and it may impact them in a way that they would understand is cathartic. It kind of allows you to let go of some of the stuﬀ that you may be holding, you know? | The process of making a digital story oﬀers participants the opportunity to release and then re-story their experience with cancer, which may allow them to make sense or meaning of their experiences   And I think it was maybe after I watched the video, I realized how beneﬁcial it was to me. You know, that it really made, well, even during the process too, it made me think about a lot of things, and I hadn’t really expressed a lot to very many people.  It sort of forces you to reﬂect on things that maybe you don’t sit down and do every day, right? It’s not like you go, “Today I’m going to think about this.” This is kind of an opportunity that forces you into something like that. It made me think about things that maybe I didn’t want to, or hadn’t very much, you know? (Jeﬀrey)  What I loved about this exercise is that it brought the most important thing out very quickly. And that now gives me more of a framework, so when people ask questions, I can talk about it within that framework. (Thomas)  And then it just showed that there was so much there that was still being processed or still being thought about. And as we went through the photos . . . it brought so much clarity to me. | Other participants described their own catharsis as “closure,” “a way of working through all this complexity,” and “a huge release.” One participant, Candice, spoke of digital stories providing a way to “do something” with her emotions:  Like, your choice really is to let [the emotions] crush you or not. And when your choice is to not, then you have no choice but to stand up and make sure you are in the best frame of mind, which means you have to do something with those emotions.  . And then, when we ﬁnished writing the story, I went through this whole, like, emotional business for a couple of days, you know? |  |  | The process of making a digital story oﬀers participants the opportunity to release and then re-story their experience with cancer, which may allow them to make sense or meaning of their experiences  [A] pregnant pause in my life . . . that makes me feel like the old me is a million miles away, and I can kind of remember her sometimes, but I don’t know if I’ll ever get back to who I was, who I used to be. But I really want to.  Yeah, before this [digital story] came, I wasn’t sharing that I had cancer or the processes that I was going through; I hadn’t explained it. So, yeah, people came forward and said, “Oh, you know, I’m seven years in remission,” or “ﬁve years in remission,” or that “I’m stage IV cancer too.” It was a really nice way to connect with others, I guess. |
| **Lamarre & Rice (2016)** |  |  | Further, their stories do not represent the outward expression of some internal feeling or experience, but another embodied experience constructed in relation, including the relations to the group and to the facilitator described above. |  |  | Margot begins her story by unpacking some of the common myths and stereotypes around eating disorders; namely, that girls who suffer from eating disorders are "shallow, vain, or fragile."  While she mentioned that she had previously questioned such destructive ideals, she highlights this as a pivotal moment in which she was able to enact a choice that led to a revisioning of what she once thought she want and what she actually wanted. As her story progresses, Margot engages in a critique of the values that fed her disorder for so long, offering a new "direction for perceiving" (GREENE, 1986, p.59) her experience.  "I no longer want to put pressure on myself to live up to the definitions of those concepts, whether externally or internally imposed. That's kind of the point." In saying "that's kind of the point," |
| **Lenette & Boddy. (2013)** | No quotations, findings described narratively but no quotations were provided. | | | | | |
| **Martin et al. (2019)** |  |  | Particularly noted was the emotional labor participants invested in revisiting painful aspects of their stories and deciding how to shape them in keeping with their current identities and hopes for their futures.  there was a wanting to leave because I’m emotionally exhausted, but wanting to stay because this is a safe, caring, and thoughtful space quality to the atmosphere |  |  |  |
| **Njeru et al. (2015)** |  | described how relieved he was to finally understand that his multiple symptoms were caused by diabetes, and how he applied his problem-solving skills in being more active and eating well. |  |  |  |  |
| **Nyirenda et al. (2022)** | On the other hand, their focus on WASH demonstrated that the DST eﬀectively empowered them to discuss priority health concerns that aﬀected them as a collective. |  |  |  | it also created a platform for participants to express their sense of powerlessness to address structural challenges leading to WASH. Opposite to gaining agency. | On the other hand, failure to use ﬁndings from DST to generate social change raise questions on the social value ofDST to community participants Counterargument. |
| **Paterno et al. (2018)** | I know what I lost with my son due to addiction, and I wanted to help the moms see that didn’t have to be their story.”   allowed me to get to know my peers on a more intimate level. Even though we worked together, there was so much we didn’t know about each other and, I mean, their videos brought me to tears, and I felt like I really got to know them.  We sit and we talk candidly with each other but to see [the digital story] up there . . . in their own voice with their own pictures, [to see] how they see their story, it was nice . . . they were beautiful . . . so that helped to bring us a little closer I think.  **be**ing able to see the finished product gave me such hope . . . for the moms of today and how I might be able to help them.”  Sarah and the other participants specifically identified the ability to use their digital stories as supportive tools to instill hope in their mentees. As another participant, Hannah, commented, “I think [for] women seeing the videos [it] might give them a sense of hope and realize that recovery is possible and you . . . there are people who are here and available and willing to support them.” | The workshop then served as a site for “doing” recovery through individual and group processes of sense-making.  I think in just the process of the digital storytelling workshop, there’s calming, there is a deeper selfknowing because you’re trying to target, you are trying to . . . have your story be understood by somebody else. And in that process, some answers come to you, too. | Having to look at myself and try to face what lies inside of me isn’t always an easy job. It’s an inside job. We have to get all that junk out of us, and then the task of trying to replace it with the beautiful begins. A transformation of sorts. |  |  |  |
| **Wexler et al. (2012)** | “I think this was a good experience for me and I think it will help many more people in the future. Because some kids need a way to express themselves.”  Thus, not only did the digital storytelling reflect positive representations of themselves and their lives, sharing their stories with important figures in participants lives also offered them a way to strengthen their relationships and build reciprocal, caring connections. | “the music and the pictures . . . brings memories back [about] how much fun [we] had in the past.” Others talked about how watching their videos made them realize how many people cared about them and how many good things they have in their lives. | While the digital storytelling process certainly caused participants to feel good by evoking positive memories, produced digital stories also served as artifacts for bolstering well-being in their daily lives. Specifically, digital stories served as mementos for participants to emphasize what was going well in their lives and especially to publicly represent the positives in their lives. |  |  | made me think of the happy memories from the pictures.”  Participants also spoke about having a sense of achievement by completing their digital stories.  noted a sense of accomplishment, saying that “getting [the digital story] done and having fun making it” was a central benefit of participating in the workshop.  It shows my life, and that we don’t live in igloos anymore. [Also, it lets others] see how much time I spend with my friends.” |
| **Willis et al. (2014)** | They stated that they would like to now train their peers to make their own digital stories; “This should be made available to everyone in Zvandiri. We know how to do it now so we can teach them” |  |  | , storytellers explained how  they now have a sense of control and freedom in their lives and how these have helped them to cope better;  “I can now take control ofmy life, I have kissed away the fear and frustration” (Susan)  “Through support, I feel conﬁdent and able to make informed choices. I am now conﬁdent, independent and most ofall ever smiling” (Tendai)  All storytellers felt that this was a process which they owned and led. “It is exciting to be the ﬁrst to try this. It truly is a project for teenagers, led by teenagers” (Precious). T  “I could really speak freely (through my own ﬁlm), about things I have never shared with anyone before” (Keith).  Participation in this process helped them to view themselves as capable young people with capacity to acquire new skills, in contrast to their previous self-perception. |  | . “You will never be able to pull me down, I can now walk alone for as many years” (Frank  “I have developed skills in copingwithmy situation. I can now look aftermyself, take steps to protectmyself” (Alan); “I can now see the opportunities given to me” (Nigel).  Participants commonly described a feeling of renewed ambition and  optimism in their lives; “My dream is to have a good job” (Brian); “Ihope to get married and have our own HIV negative children” (Rudo). To illustrate her ambition, one girl chose to include a visual image of her dressed as a nurse in her HIV clinic.  In contrast to the earlier feelings of hopelessness, suicidal ideation and having no purpose, they then went on to describe a new desire to live; “I now have the desire to live…hope for a brighter future”  (Alan). “I will achieve my goals because I have a positive mind” (Lindiwe). “My life has been a rollercoaster,(but is now) going from strength to good health” (Susan) |

**Table 7: Second Iteration of the Framework**

The health-promoting experiences of storytellers participating in group-based digital storytelling workshops (Meta-synthesis, Switzerland, 2024).

| **Descriptive Themes (Review Findings)** | **Studies contributing to the review finding** | **Analytical theme** |
| --- | --- | --- |
| **Overcoming vulnerability** – Entering the DST space often generates initial fears and apprehensions triggered by the prospect of sharing sensitive and intimate stories with strangers. Storytellers fear being judged or misunderstood and grapple with the uncertainty of how their stories unfold. Storytellers cautiously navigate the DST process, but this sense of vulnerability dissipates as they share their stories. Storytellers recognize their peers´ bravery, inspiring them to share their truths and reinforcing the belief that their stories must be told. | **11 Studies**  [11, 55-57, 87-93] | **Narrative shift** |
| **Deliberate sense-making—**Storytellers make sense of their experiences as they write their scripts, collect visual and auditory material to illustrate their stories, and share them with fellow storytellers. This process provides a space for deliberate reflection, finding meaning from their experiences, and capturing their emotional state. Some storytellers find new meaning in their experiences and value the opportunity to learn more about themselves as individuals and community members through this deliberate process. | **14 Studies**  [5, 11, 15, 56, 57, 87, 90, 92, 94-99] |  |
| **Shaping or replacing existing narratives—** Storytellers undergo a shift in perspective as they construct their stories, resulting in the altering, reshaping, or even replacing of the narratives they initially bring into the DST process about their lived experiences. Storytellers uncover previously unknown connections to other aspects of their lives by re-examining their own beliefs, perspectives, and lived experiences as they listen to the stories of others. Some storytellers view it as an opportunity to challenge and correct existing, socially accepted narratives and discourses and as an accomplishment. | **16 Studies**  [5, 11, 15, 56, 57, 87, 88, 92-96, 99-102] |  |
| **Sharing and connecting in a safe space**—The digital storytelling process creates a safe and supportive space, an environment of mutual respect and understanding, where storytellers are empowered to speak freely about their experiences of health, even about their most guarded secrets, as they develop a sense of connection with other storytellers. | **7 Studies**  [5, 11, 56, 57, 94, 98, 103] | **The ripple effect of digital storytelling – waves of empathy, understanding and connection** |
| **From empathy to compassion**: Listening to other storytellers' stories generates an empathetic response driven by learning about their shared experiences, motivating storytellers to act and mutually support each other. Some storytellers gain an awareness that they are part of something bigger and feel that their stories can help both fellow storytellers and those beyond the digital storytelling workshop. They feel compelled to tell their stories to help others. | **14 Studies**  [5, 11, 56, 57, 87, 88, 91, 92, 94, 95, 98, 99, 102, 103] |  |
| **Sharing stories brings comfort** - During the digital storytelling process, learning that you are not alone can be comforting. Knowing that others have similar experiences validates the storytellers' feelings and emotions, and storytellers find common ground in their experiences, generating a sense of collective hope and unity. | **8 Studies**  [5, 15, 55-57, 87, 90, 94] |  |
| **Increased Sense of Community Belonging** – Learning that others go through similar situations generates a strong bond between the storytellers that inspires a commitment beyond the confines of the workshop. Storytellers demonstrate a commitment to sustaining and growing their newfound community. | **10 Studies**  [5, 11, 56, 57, 90, 93, 94, 98, 99, 103] |  |
| **Experiencing emotional resonance**—The digital stories portray emotions and feelings that strike a chord with the storytellers. As storytellers watch and listen to the stories, they recognise and connect with these emotions as they realise they share similar emotional experiences. | **8 Studies**  [5, 11, 56, 57, 90, 92, 96, 104] | **Investing and processing emotions for healing** |
| **Harnessing the amplified emotions—**Triggered by the evoked emotional resonance, storytellers experience a heightened emotional awareness as they write and produce their digital stories and listen to the stories of others. This is experienced as an opportunity to work and process these emotional burdens and open up to sharing things that otherwise, in other contexts, they would not share. The emotions drive storytellers to construct stories that portray "the rawness of these emotions". | **8 Studies**  [5, 57, 88, 92, 95, 96, 98, 103, 104] |  |
|  | **11 Studies**  [5, 15, 56, 57, 87, 88, 90, 92, 94, 95, 101] |  |
| **Coming to terms with experiences of illness or trauma**— Storytellers experience a sense of relief from their emotional burdens, feel liberated from past memories of adverse experiences, and feel at peace, having shared their stories with fellow storytellers who understand their pain and suffering. Some storytellers refer to the process as therapeutic and cathartic because it helps them confront and process their emotions and feel healed, having achieved some form of personal growth. | **8 Studies**  [5, 11, 56, 57, 90, 92, 96, 104] |  |
| **Increased sense of control over their experiences—**As Storytellers construct their stories, they go through a process of self-discovery that appears to build their confidence. They feel that they have some level of influence or power over their circumstances. This is amplified by the collaborative approach to story development, which grants them a sense of empowerment that moves them to create stories that either challenge existing narratives and discourses about their lived experiences or send a message to help others. | **13 Studies**  [5, 15, 55-57, 87, 88, 92, 94, 95, 98, 102, 103] | **Steering the narrative through self-discovery** |
| **Gaining Agency**—After completing the digital story, storytellers recognise that the stories can potentially resonate with others. They acquire a sense of purpose and express their desire to use their stories to improve the lives of those with similar experiences. | **8 Studies**  [5, 56, 57, 92, 94, 96, 98, 102] |  |
