# Supplementary material for: The health-promoting experiences of storytellers in group-based digital storytelling workshops: a meta-synthesis of qualitative studies
Source: Front Digit Health. 2025 Oct 29;7:1607897. doi: 10.3389/fdgth.2025.1607897 (PMC12605066; doi:10.3389/fdgth.2025.1607897)
Supplement: Supplementary file 1 [file Datasheet1.zip › Appendix 3.docx]

**Appendix 3:**

**Table 8: Evidence profile table GRADECerqual** The health-promoting experiences of storytellers participating in group-based digital storytelling workshops (Meta-synthesis, Switzerland, 2024).

| **#** | **Summarised review finding** | **Methodological limitations** | **Coherence** | **Adequacy** | **Relevance** | **GRADE-CERQual Overall assessment of confidence** | **References** |
| --- | --- | --- | --- | --- | --- | --- | --- |
| 1 | **Overcoming vulnerability** – Entering the DST space often generates initial fears and apprehensions triggered by the prospect of sharing sensitive and intimate stories with strangers. Storytellers fear being judged or misunderstood and grapple with the uncertainty of how their stories unfold. Storytellers cautiously navigate the DST process, but this sense of vulnerability dissipates as they share their stories. Storytellers recognise their peers´ bravery, inspiring them to share their truths and reinforcing the belief that their stories must be told. | **Minor concerns**  **Explanation:** There are minor concerns regarding methodological limitations because recruitment strategies used by some of the included studies may have introduced biases. For instance, some participants had to participate in the research as part of their further education at the organisation they belonged to. In addition, only some of the studies consider aspects of reflexivity. | **No/Very minor concerns**  **Explanation:** All the included studies provide data to some extent as to how the storytellers enter the workshop with some degree of anxiety. | **Minor concerns**  **Explanation:** Minor concerns regarding adequacy exist because although 11 studies provided data for this review finding, some of the data provided is thin and limited to the observations of a single participant or, in some cases, two participants. However, these concerns are minor because the prevalence of this finding across the relevant studies is high, even though it is not rich. In addition, the finding is somewhat descriptive, thus requiring lower level of data richness. | **Moderate concerns**  **Explanation:** There are moderate concerns regarding relevance because the data of some studies where this finding emanates is partially relevant to the review question. Whilst all use a digital storytelling approach, their main focus is not on the experience of the storytellers but on exploring the phenomenon of interest, including the experiences of living with a particular illness of having been exposed to some trauma. | **Moderate confidence**  **Explanation:** Minor concerns regarding methodological limitations, No/Very minor concerns regarding coherence, Minor concerns regarding adequacy, and Moderate concerns regarding relevance | Boydell et al. 2018; De Vecchi et al. 2017; DiFulvio et al. 2016; Fiddian-Green et al. 2017; Gubrium et al. 2016; Gubrium et al. 2019; Howard et al. 2023; Jun et al. 2022; Kim et al. 2023; Laing et al. 2017; Nyirenda et al. 2022; |
| 2 | **Deliberate sense-making**—Storytellers make sense of their experiences as they write their scripts, collect visual and auditory material to illustrate their stories and share them with fellow storytellers. This process provides a space for deliberate reflection, finding meaning from their experiences, and capturing their emotional state. Some storytellers find new meaning in their experiences and value the opportunity to learn more about themselves as individuals and community members through this deliberate process. | **Serious concerns**  **Explanation:** Serious concerns regarding methodological limitations because most included studies failed to address the relationship between researchers and participants. Some studies did not provide solid evidence as to how the data analysis was conducted. Recruitment through third parties was also used in some of the studies. | **No/Very minor concerns**  **Explanation:** Whilst this is an explanatory finding, most studies refer to the process of sense-making. Thus, the degree of transformation is close to the thematic description. | **No/Very minor concerns**  **Explanation:** The data is rich (there is more than just one quotation), and 14 studies explicitly mention the active sense-making process guided by the facilitation of the DST. | **Minor concerns**  **Explanation:** Minor concerns regarding relevance because some of the included studies did not seek to actually review the experiences of storytellers. However, the data provide sufficient information to reflect this finding. | **Moderate confidence**  **Explanation:** Serious concerns regarding methodological limitations, No/Very minor concerns regarding coherence, No/Very minor concerns regarding adequacy, and Minor concerns regarding relevance. The methodological quality of some of the included studies is low. There is a need to consider that this introduces serious concerns about the finding. However, the data is rich and many studies provide evidence of this finding. | Beltrán & Begun 2014; Boydell et al. 2018; Briant et al. 2016; De Vecchi et al. 2017; Ferrari M et al. 2015; Goodman 2019; Gubrium et al. 2016; Howard et al. 2023; Jun et al. 2022; Laing et al. 2017; Laing et al. 2019; Njeru et al. 2015; Paterno et al. 2018; Wexler et al. 2013; |
| 3 | **Shaping or replacing existing narratives**— Some storytellers undergo a profound shift in perspective as they construct their stories, resulting in the altering, reshaping, or even replacing of the narratives they initially brought into the DST process about their lived experiences. Storytellers uncover previously unknown connections to other aspects of their lives by re-examining their own beliefs, perspectives, and lived experiences as they listen to the stories of others. Some storytellers view it as an opportunity to challenge and correct existing, socially accepted narratives and discourses and as an accomplishment. | **Serious concerns**  **Explanation:** Serious concerns regarding methodological limitations because most of the included studies reflecting this finding did not address reflexive issues, had limited descriptions of their data analysis, did not provide a clear aim or assessing their recruitment strategy was not possible due to limited information. The lack of detail, makes it uncertain and, thus increases the concerns as to the methodological rigour of the included studies. | **Minor concerns**  **Explanation:** Minor concerns regarding coherence because there was a higher degree of transformation. The data reflect, however, a change and a sense of accomplishment. Thus, there also descriptive findings that support the review finding. | **Minor concerns**  **Explanation:** Minor concerns regarding adequacy because the data is thinner. Two studies contribute the most to this finding (Laing et al. 2017; Laing et al. 2019;). While they provide richer data, the other studies demonstrated less prominent data. However, 16 studies provide some data for this finding., which increases its prevalence among the included studies. | **Minor concerns**  **Explanation:** Minor concerns regarding relevance because the studies addressing this finding are situated within the health and digital storytelling context. The studies address the research question, but some are not addressing a clear health illness. | **Low confidence**  **Explanation:** Serious concerns regarding methodological limitations, Minor concerns regarding coherence, Minor concerns regarding adequacy, and Minor concerns regarding relevance. The lack of methodological rigour, combined with the richness of the data and its transformation, raise some concerns regarding its representation of the experiences of storytellers. | Beltrán & Begun 2014; Boydell et al. 2018; Briant et al. 2016; De Vecchi et al. 2017; DiFulvio et al. 2016; Dixon & Isaac 2023; Ferrari M et al. 2015; Goodman 2019; Gubrium et al. 2016; Howard et al. 2023; Laing et al. 2017; Laing et al. 2019; Lamarre & Rice 2016; Nyirenda et al. 2022; Wexler et al. 2013; Willis et al. 2014; |
| 4 | **Sharing and connecting in a safe space**—The DST process creates a safe and supportive space, an environment of mutual respect and understanding, where storytellers are empowered to speak freely about their experiences, even about their most guarded secrets, as they develop a sense of connection with other storytellers. | **Moderate concerns**  **Explanation:** Moderate concerns regarding methodological limitations because most studies do not address the researcher´s relationship with the participants. There is a lack of detail concerning data analysis in some of the studies. However, two of the included studies do not demonstrate high methodological rigour. | **No/Very minor concerns**  **Explanation:** A low degree of data transformation results in a more descriptive finding. The data explicitly refer to this finding, demonstrating a high level of coherence. | **Moderate concerns**  **Explanation:** Moderate concerns regarding adequacy because the data is thinner and only a few studies refer to this finding. However, there is enough data describing this finding for it being considered. | **Minor concerns**  **Explanation:** Minor concerns regarding relevance because whilst most studies are contextually placed within the scope of the research question, some of them provide indirect evidence for his findings, focusing on their phenomenon of interest and not the experiences of storytellers. | **Moderate confidence**  **Explanation:** Moderate concerns regarding methodological limitations, No/Very minor concerns regarding coherence, Moderate concerns regarding adequacy, and Minor concerns regarding relevance. Despite the methodological limitations of the included studies, these are not strong and could have impacted the findings dramatically. The reduced adequacy raises concern, but the high coherence and relevance make a judgement of moderate confidence suitable. | Beltrán & Begun 2014; Briant et al. 2016; De Vecchi et al. 2017; Gubrium et al. 2016; Howard et al. 2023; Kim et al. 2021; Paterno et al. 2018; |
| 5 | **From empathy to compassion-** Listening to other storytellers' stories generates an empathetic response driven by learning about their shared experiences, motivating storytellers to act and mutually support each other. Some storytellers gain an awareness that they are part of something bigger and feel that their stories can help both fellow storytellers and those beyond the DST workshop. They feel compelled to tell their stories to help others. | **Serious concerns**  **Explanation:** There are serious concerns regarding methodological limitations because whilst two studies did not show methodological issues, the rest of the studies addressing this finding lack methodological rigour. This refers to a lack of analytical reflwxivity, not describing in detail the process of data analysis and some issues with sample selection. | **No/Very minor concerns**  **Explanation:** The finding is coherent with the data. There has been a high degree of transformation in the development of this finding. However, there are also explicit statements that reflect the finding. | **No/Very minor concerns**  **Explanation:** This was a very prominent data item. Whilst some of the included studies contributed more than others, the data is rich and highly prevalent. | **Moderate concerns**  **Explanation:** There are moderate concerns regarding relevance because some studies provide indirect evidence. Some of the studies focused on their phenomenon of interest and not on the experiences of the storytellers. The context of the three studies is related but beyond the health-related outcomes of participating in a DST process. | **Moderate confidence**  **Explanation:** Serious concerns regarding methodological limitations, No/Very minor concerns regarding coherence, No/Very minor concerns regarding adequacy, and Moderate concerns regarding relevance. The lack of methodological rigour of most included studies and the indirect evidence due to contextual differences raised some concerns. | Beltrán & Begun 2014; Boydell et al. 2018; Briant et al. 2016; De Vecchi et al. 2017; DiFulvio et al. 2016; Goodman 2019; Gubrium et al. 2016; Howard et al. 2023; Kim et al. 2021; Kim et al. 2023; Laing et al. 2017; Paterno et al. 2018; Wexler et al. 2013; Willis et al. 2014; |
| 6 | **Sharing stories brings comfort** - During the DST process, learning that you are not alone can be comforting. Knowing that others have similar experiences validates the storytellers' feelings and emotions and storytellers find common ground in their experiences, cultivating a sense of collective hope and unity. | **Moderate concerns**  **Explanation:** Moderate concerns regarding methodological limitations because of a lack of reflexivity in most of the included studies. however, three studies demonstrated no methodological issues. There is also issues with recruitment and a lack of the analytical process followed. | **No/Very minor concerns**  **Explanation:** There is a degree of transformation of the data into the finding. However, it is coherent. In some cases there is a explicit statement about not being alone. | **Minor concerns**  **Explanation:** There are minor concerns regarding adequacy because the data is less rich and less prevalent in the included studies. Given the transformation of the finding into a more explanation finding, richer data would be more suitable. | **Minor concerns**  **Explanation:** Minor concerns regarding relevance because there are some contextual differences from the review question and some studies provide indirect evidence. | **Moderate confidence**  **Explanation:** Moderate concerns regarding methodological limitations, No/Very minor concerns regarding coherence, Minor concerns regarding adequacy, and Minor concerns regarding relevance. Lack of methodological rigour and adequacy requires a downgrade of the confidence in this finding. | Beltrán & Begun 2014; Boydell et al. 2018; Briant et al. 2016; Ferrari M et al. 2015; Gubrium et al. 2016; Gubrium et al. 2019; Howard et al. 2023; Jun et al. 2022; |
| 7 | **Increased Sense of Community Belonging** – Learning that others go through similar situations generated a strong bond between the storytellers that inspired a commitment beyond the confines of the workshop. Some storytellers express a desire to remain connected, demonstrating a commitment to sustaining and growing their newfound community. | **Moderate concerns**  **Explanation:** Moderate concerns regarding methodological limitations because only two studies are free from limitations. A lack of reflexivity. The analytical process of some of the included studies does not provide enough detail to judge the adequacy of the analysis. One of the studies showed serious limitations, while others failed to state the study's objectives clearly. | **No/Very minor concerns**  **Explanation:** This is a descriptive finding. The data is coherent with the finding. There are explicit expressions of the sense of community gained through DST. | **No/Very minor concerns**  **Explanation:** The data is rich. All but one study provide several items of data reflecting this finding. This finding is prevalent and plausible. | **Minor concerns**  **Explanation:** Minor concerns regarding relevance because not all the studies relevant to this finding, are within the health context and focus on the phenomenon of interests and not on the experiences of storytellers. | **High confidence**  **Explanation:** Moderate concerns regarding methodological limitations, No/Very minor concerns regarding coherence, No/Very minor concerns regarding adequacy, and Minor concerns regarding relevance. Despite the lack of rigour in some of the included studies, this finding is very prevalent and backed by rich evidence that is found more than once in the studies. | Beltrán & Begun 2014; Briant et al. 2016; De Vecchi et al. 2017; Gubrium et al. 2016; Howard et al. 2023; Jun et al. 2022; Kim et al. 2021; Nyirenda et al. 2022; Paterno et al. 2018; Wexler et al. 2013; |
| 8 | **Experiencing emotional resonance**—The digital stories portray emotions and feelings that strike a chord with the storytellers. As storytellers watch and listen to the stories, they recognise and connect with these emotions as they realise they share similar emotional experiences. | **Moderate concerns**  **Explanation:** Moderate concerns regarding methodological limitations because of issues with reflexivity and a lack of detail about data analysis that did not allow a judgement on rigour. | **Minor concerns**  **Explanation:** Minor concerns regarding coherence because this finding reflects a higher degree of transformation. Storytellers reflect their emotional journey, but some data items are less coherent with the finding. However, there is also explicit quotations that support this finding. | **Moderate concerns**  **Explanation:** Moderate concerns regarding adequacy because the data is thinner and is only reflected in 8 studies. Given the degree of transformation, turning this finding into a more explanatory finding, there is a need for richer and more prevalent data. Nonetheless, there is enough data to arrive at this finding. | **Minor concerns**  **Explanation:** Minor concerns regarding relevance because two of the included studies reviewed issues of traumatic experiences that, whilst not directly a health concern, can lead to mental illness. However,most studies focused on the experiences of storytellers and thus, provide direct evidence to support this finding. | **Moderate confidence**  **Explanation:** Moderate concerns regarding methodological limitations, Minor concerns regarding coherence, Moderate concerns regarding adequacy, and Minor concerns regarding relevance. Issues with rigour and thinner data items bring the confidence in this finding a level down. | Beltrán & Begun 2014; De Vecchi et al. 2017; Gubrium et al. 2016; Howard et al. 2023; Jun et al. 2022; Laing et al. 2017; Laing et al. 2019; Martin et al. 2019; |
| 9 | **Harnessing the amplified emotions**—Triggered by the evoked emotional resonance, storytellers experience a heightened emotional awareness as they write and produce their digital stories and listen to the stories of others. This is experienced as an opportunity to work and process these emotional burdens and open up to sharing things that otherwise, in other contexts, they would not share. The emotions drive storytellers to construct stories that portray "the rawness of these emotions". | **Moderate concerns**  **Explanation:** Moderate concerns regarding methodological limitations because of lack of reflexivity and a lack detail with regard to the analytic approach. | **No/Very minor concerns**  **Explanation:** This is a very explnantory finding with a high degree of transformation. There is coherence in the data referring to a heightened level of emotional awareness. | **Minor concerns**  **Explanation:** Minor concerns regarding adequacy because whilst the data is prevalent in the included studies, and there a re more than a data item in most studies, the data is not that rich. Given the level of transformation, richer data would have resulted in no concerns. However, there is enough data to warrant a minor concerns judgment. | **Minor concerns**  **Explanation:** Minor concerns regarding relevance because some of the evidence is indirect due to contextual differences and the fact that not all studies focused on storytellers experiences- | **Moderate confidence**  **Explanation:** Moderate concerns regarding methodological limitations, No/Very minor concerns regarding coherence, Minor concerns regarding adequacy, and Minor concerns regarding relevance. Rigour and adequacy raise concerns and thus, the lower level of confidence. | Beltrán & Begun 2014; DiFulvio et al. 2016; Goodman 2019; Howard et al. 2023; Kim et al. 2021; Laing et al. 2017; Laing et al. 2019; Martin et al. 2019; Paterno et al. 2018; |
| 10 | **Coming to terms with experiences of illness or trauma— Storytellers** experience a sense of relief from their emotional burdens, feel liberated from past memories of adverse experiences, and feel at peace, having shared their stories with fellow storytellers who understand their pain and suffering. Some storytellers refer to the process as therapeutic and cathartic because it helps them confront and process their emotions and feel healed, having achieved some form of personal growth. | **Moderate concerns**  **Explanation:** There are moderate concerns regarding methodological limitations because the studies did not explain the researcher's relationship with the storytellers, and some of them did not provide enough detail about the analytic process. Lack of detail regarding recruitment and not providing a clear aim. | **No/Very minor concerns**  **Explanation:** There is a strong coherence between the data and the review finding. There has been a lower degree of transformation. Storytellers reflect the benefits of telling their stories to peers. | **No/Very minor concerns**  **Explanation:** The data is rich and prevalent in most relevant studies. | **Minor concerns**  **Explanation:** Minor concerns regarding relevance because some of the studies focus on the phenomenon of interest and not on the storytellers experiences. | **Moderate confidence**  **Explanation:** Moderate concerns regarding methodological limitations, No/Very minor concerns regarding coherence, No/Very minor concerns regarding adequacy, and Minor concerns regarding relevance. there is enough evidence to have a moderate confidence, but due to the issues wth rigour, it requires a downgrading of the evidence. | Beltrán & Begun 2014; Boydell et al. 2018; Briant et al. 2016; DiFulvio et al. 2016; Ferrari M et al. 2015; Goodman 2019; Gubrium et al. 2016; Howard et al. 2023; Jun et al. 2022; Laing et al. 2017; Lamarre & Rice 2016; |
| 11 | **Increased sense of control over their experiences**—As Storytellers construct their stories, they go through a process of self-discovery that appears to build their confidence. They feel that they have some level of influence or power over their circumstances. This is amplified by the collaborative approach to story development, which grants them a sense of empowerment that moves them to create stories that either challenge existing narratives and discourses about their lived experiences or send a message to help others. | **Moderate concerns**  **Explanation:** Moderate concerns regarding methodological limitations because due a lack of reflexivity from most included studies, some issues with the data analysis process and a lack of detail about recruitment strategies, study aims, ethical considerations and appropriateness of qualitative design. | **No/Very minor concerns**  **Explanation:** The data has been transformed but remains somewhat descriptive. Thus, there is a high level of coherence between the data and the review finding. | **No/Very minor concerns**  **Explanation:** The data is rich and many studies reflected the review finding. There is enough evidence in the data for even a more explanatory finding. | **Minor concerns**  **Explanation:** Minor concerns regarding relevance because not all studies address storytellers' experiences and, thus, provide secondary evidence to this review finding. However, since some of the included studies address this finding explicitly, there are only minor concerns. | **High confidence**  **Explanation:** Moderate concerns regarding methodological limitations, No/Very minor concerns regarding coherence, No/Very minor concerns regarding adequacy, and Minor concerns regarding relevance. Despite the methodological limitations, there is enough rich evidence to have a higher level of confidence. | Beltrán & Begun 2014; Boydell et al. 2018; Briant et al. 2016; DiFulvio et al. 2016; Ferrari M et al. 2015; Goodman 2019; Gubrium et al. 2016; Gubrium et al. 2019; Howard et al. 2023; Kim et al. 2021; Laing et al. 2017; Paterno et al. 2018; Willis et al. 2014; |
| 12 | **Gaining Agency**—After completing the digital story, storytellers recognise that the stories can potentially resonate with others. They acquire a sense of purpose and express their desire to use their stories to improve the lives of those with similar experiences. | **Moderate concerns**  **Explanation:** Moderate concerns regarding methodological limitations because of a lack of addressing the researcher's position in the research and some lack of detail with regard to analysis. | **Minor concerns**  **Explanation:** Minor concerns regarding coherence because some of the data require a high level of interpretation. However, there is coherence between most data from the relevant studies. This only minor concerns. | **Minor concerns**  **Explanation:** Minor concerns regarding adequacy because the data is less prevalent. In addition, given the degree of explanation of this finding, richer data would have been necessary to avoid the downgrade. | **Minor concerns**  **Explanation:** Minor concerns regarding relevance because of contextual differences between the studies that do not address the experiences of storytellers. In addition, there is a strong focus on the relevant studies on the phenomenon of interest. | **Moderate confidence**  **Explanation:** Moderate concerns regarding methodological limitations, Minor concerns regarding coherence, Minor concerns regarding adequacy, and Minor concerns regarding relevance. The methodological issues and the limited data provided by the studies, limit the confidence in this review finding. | Beltrán & Begun 2014; Briant et al. 2016; Gubrium et al. 2016; Howard et al. 2023; Laing et al. 2017; Laing et al. 2019; Paterno et al. 2018; Willis et al. 2014; |
